# Genome-wide computational analysis of potential long noncoding RNA mediated DNA:DNA:RNA triplexes in the human genome

**DOI:** 10.1186/s12967-017-1282-9

**Published:** 2017-09-02

**Authors:** Saakshi Jalali, Amrita Singh, Souvik Maiti, Vinod Scaria

**Affiliations:** 1 CSIR Institute of Genomics and Integrative Biology (CSIR-IGIB), Mathura Road, Delhi, 110020 India; 2 Academy of Scientific and Innovative Research (AcSIR), CSIR IGIB South Campus, Mathura Road, Delhi, 110020 India

**Keywords:** Long non coding RNAs, Triplex forming sites, RNA–DNA interactions, Triple-helix

## Abstract

**Background:**

Only a handful of long noncoding RNAs have been functionally characterized. They are known to modulate regulation through interacting with other biomolecules in the cell: DNA, RNA and protein. Though there have been detailed investigations on lncRNA-miRNA and lncRNA-protein interactions, the interaction of lncRNAs with DNA have not been studied extensively. In the present study, we explore whether lncRNAs could modulate genomic regulation by interacting with DNA through the formation of highly stable DNA:DNA:RNA triplexes.

**Methods:**

We computationally screened 23,898 lncRNA transcripts as annotated by GENCODE, across the human genome for potential triplex forming sequence stretches (PTS). The PTS frequencies were compared across 5′UTR, CDS, 3′UTR, introns, promoter and 1000 bases downstream of the transcription termination sites. These regions were annotated by mapping to experimental regulatory regions, classes of repeat regions and transcription factors. We validated few putative triplex mediated interactions where lncRNA-gene pair interaction is via pyrimidine triplex motif using biophysical methods.

**Results:**

We identified 20,04,034 PTS sites to be enriched in promoter and intronic regions across human genome. Additional analysis of the association of PTS with core promoter elements revealed a systematic paucity of PTS in all regulatory regions, except TF binding sites. A total of 25 transcription factors were found to be associated with PTS. Using an interaction network, we showed that a subset of the triplex forming lncRNAs, have a positive association with gene promoters. We also demonstrated an in vitro interaction of one lncRNA candidate with its predicted gene target promoter regions.

**Conclusions:**

Our analysis shows that PTS are enriched in gene promoter and largely associated with simple repeats. The current study suggests a major role of a subset of lncRNAs in mediating chromatin organization modulation through CTCF and NSRF proteins.

**Electronic supplementary material:**

The online version of this article (doi:10.1186/s12967-017-1282-9) contains supplementary material, which is available to authorized users.

## Background

Developments in the field of transcriptomics contributed to the discovery of vast number of RNAs, majority of which are non-coding RNAs [1]. Long non-coding RNAs though do not code for functional proteins, but are known to play significant regulatory roles which in turn impact various biological processes including development, differentiation, and metabolism [2–5]. While only a small number of the lncRNAs have been extensively characterized, they are largely thought to act by their interaction with other biomolecules in the cell: DNA, RNA and protein [6–10]. However, lncRNA-miRNA and lncRNA-protein interactions have been discussed previously [7, 11, 12], the interaction of lncRNAs with DNA has not been studied extensively [13]. Previous studies have reported that binding sites of well-known lncRNA HOTAIR and HOTTIP have an enriched DNA sequence motif. Using chromatin isolation by RNA purification sequencing (ChIRP-seq) technique, it was revealed HOTAIR lncRNA preferentially occupies a GA-rich DNA motif leading to disruption in Polycomb occupancy thereby regulating the chromatin state [14]. Alternatively, Xist does not directly interact with DNA, rather harnesses the sequence-specific YY1 transcription factor to attach to sites in the X chromosome [14, 15]. The lncRNA Fendrr expressing in lateral mesoderm of mid-gestational mouse embryos, interacts with both PRC2 and TrxG/Mll complexes in vivo via dsDNA/RNA triplex formation at target regulatory elements, and directly increases PRC2 occupancy at these sites [16]. In another example, the region between minor and major transcript initiation sites (~400 bp) of DHFR gene gives rise to a noncoding RNA transcript. This lncRNA transcript has shown to repress the transcription of downstream protein coding gene by forming a purine–purine–pyrimidine triplex motif with the DHFR promoter [17, 18].

PARTICLE lncRNA that expresses after low dose radiation exposure was reported to interact with the tumor suppressor methionine adenosyltransferase (MAT2A) promoter through triple helix formation. It was also observed to interact with transcription-repressive complex proteins G9a and SUZ12 (subunit of PRC2) and cause transcriptional repression of MAT2A gene via methylation of this promoter [19]. Recent reports show presence of GA-rich sequences at MEG3 binding sites, which modulate the interaction of the lncRNA through RNA–DNA triplex formation, and is assumed to be a characteristic of target gene recognition by the chromatin-interacting lncRNAs [20]. Another similar report, depicts RNA:DNA–DNA triplex formation at *SPHK1* (Sphingosine kinase 1) promoter by an antisense RNA khps1. This leads to up regulation of the *SPHK1* gene via histone acetylation and ultimately cause increase in cell proliferation [21]. Thus it can be interpreted that lncRNAs could function through interactions with genomic DNA by forming of DNA–RNA triplexes, where lncRNAs act as a third strand [7].

Since long time intermolecular triple helix formation has been implicated as possible mechanism of controlling cellular processes such as inhibiting protein-DNA interaction and functional processes including transcriptional regulation, chromatin modification, post-transcriptional RNA processing and DNA repair which is mainly revealed by in vitro experiments [22]. Large number of proteins such as helicases, heterogeneous ribonucleoproteins (hnRNP), cytoplasmic type III intermediate filament (IF), transcription factors (TFs), high mobility group (HMG) box proteins as well as proteins involved in the cell cycle and DNA repair have shown to be associated via interacting at the triplex sites [23]. Traditionally the presence of triplexes in vitro was investigated by gel retardation assays, circular dichroism and UV absorbance spectroscopy. Gel shift in gel retardation assay, presence of two melting peaks and a sharp negative peak at 210 nm in UV melting and CD spectroscopy respectively are characteristic features that helps in detection and validation of triple helical structures in vitro [22, 24]. A number of experimental approaches to identify RNA–DNA interaction sites have been used in the recent years such as chromatin isolation by RNA purification (ChIRP) [25] and capture hybridization analysis of RNA targets (CHART) [26]. These approaches are based on affinity capture of target RNA: chromatin complex or RNA by designing antisense-oligos, followed by high-throughput sequencing which then produces a map of genomic binding sites. Computational approaches for instance, Triplexator [27], R-loop finder [28] and Triplex-Inspector [29] offers enormous promises to computationally predict such interactions on genome-scale. Thus the upcoming methodologies to discover triplexes in the genome can aid in the study of functional triplexes and the roles played by non-coding RNAs.

Depending on sequence composition and relative orientation of the RNA strand (i.e. third strand interacting with duplex DNA), triplex structures can form 3 types of motifs (1) pyrimidine motif (Y) wherein the third strand is composed of pyrimidine (CT) bases bound parallel to the purine strand of DNA (2) purine motif (R) wherein the third strand is composed of purine (AG) bases bound antiparallel to the purine strand of DNA (3) mixed motif (M) where guanines and thymines bind either parallel or anti-parallel with respect to the purines in the duplex [24, 27]. Using an exhaustive computational approach, we screened the human genome for potential triplexes mediated through lncRNAs to understand their possible function mediated through formation of a triplex.

In the present study, we explore evidence on whether lncRNAs could modulate genomic regulation by interacting with DNA through the formation of highly stable DNA: DNA: RNA triplexes. The enrichment of PTS in the promoters of genes suggests its role in gene regulation and the same was evident when we constructed a co-relation network between lncRNAs and genes, consistent with the known role of some lncRNAs in transcriptional and epigenetic regulation of genes. To the best of our knowledge, this is the first comprehensive genome-wide computational analysis of PTS mediated through lncRNAs.

## Methods

### Datasets

#### Sequences

The long noncoding RNA sequences were downloaded from the GENCODE database. The sequences corresponded to the v19 release of the GENCODE. The dataset contained sequences for 23,898 long noncoding RNA transcripts corresponding to 13,853 lncRNA genes as annotated by GENCODE. The reference genome sequence was downloaded from the UCSC Genome browser and corresponds to hg19 build of the human genome.

#### Genomic positions

The Genomic positions corresponding to 1000 bases upstream of the TSS (promoter), 5′ Untranslated regions (5′UTR), Coding Exons (CDS), Introns, 3′ Untranslated Regions (3′UTR) and 1000 bp downstream of TES (1000 bp down) were downloaded in BED format from UCSC Genome Browser using the Table export feature.

#### Regulatory regions and annotations

A number of experimental tracks on regulatory regions in the genome were downloaded. We retrieved the tracks corresponding to CpG islands, Histone modifications (H3K4Me1, H3K4me3 and H3K27Ac), Transcription Factor ChIP datasets, DNAase-I Hypersensitive sites, Literature Curated regulatory regions (Open Regulatory Annotations: ORegAnno) and Enhancers from the UCSC Genome browser using the Table export feature.

#### Repeat regions

Datasets for ten different classes of repeats including DNA repeat elements (DRE), long interspersed nuclear elements (LINE), low complexity repeats (LCR), long terminal repeat elements (LTR), rolling circle (RC), RNA Repeats (RR), Satellite (Sa), short interspersed nuclear elements (SINE), simple repeats (SR), interrupted repeats (IR) and unknown (UC) were retrieved from UCSC Genome browser.

#### Housekeeping and tissue specific genes

A dataset of 2064 housekeeping genes and 2293 tissue specific genes were derived from a recent publication which performed meta-analysis of microarray datasets [30].

#### Transcription factor binding sites

The data for transcription factor was derived in form of peaks using UCSC table browser option. The data was in form of bed file. The transcription factor binding sites (TFBS) were overlapped with potential triplex forming site (PTS) in duplex DNA sites keeping the criteria as the TFBS should exactly fall/lie within the PTS sites. The overlap with the different genomic segments was performed using IntersectBED option IN BEDtools. The plots against the TSS/TES were plotted using NGSplot.

#### Hi-C interaction domains

The Hi-C (high-throughput chromosome conformation capture) data in form of bed files for 13 datasets was downloaded from http://egg.wustl.edu/d/hg19/. These processed datasets corresponding to four cell-lines was used to overlap with the PTS sites using BEDtools IntersectBED option. The Hi-C data in from of peaks was mapped on the PTS sites such that they completely lie within the PTS sites.

### Computational analysis

#### Triplex forming lncRNA predictions

The potential DNA:DNA:RNA triplex sites were predicted using Triplexator [http://bioinformatics.org.au/tools/triplexator/manual.html]. The predictions were performed genome wide for each of the human chromosome independently keeping the parameters as -l 35 -e 10 -g 20 -m R/Y/M -fm 0 -of 1 -fr off (where l: lower length bound, e: error rate less than 10%, g: guanine rate less than equal to 20%, m: type of triplex motif; R: purine motif, Y: pyrimidine motif, M: purine–pyrimidine motif, fr: filter repeats, of: output format). Additional file 1 provides the details of parameters used in our study. In addition, we also predicted the well-known triplex forming lnRNAs at different parameters using Triplexator viz. MALAT1 (as -l 35/40 -e 20/20 -g 20 -m R/Y/M -fm 0 -of 1 -fr off), HOTAIR (as -l 35/40 -e 20/20 -g 20 -m R/Y/M -fm 0 -of 1 -fr off), FENDRR (as -l 35 -e 20 -g 20 -m R/Y/M -fm 0 -of 1 -fr off) and DHFR (as -l 10 -e 10 -g 20 -m R/Y/M -fm 0 -of 1 -fr off). The list of these four lncRNAs at various parameters predicted from our analysis is given Additional file 2. The complete list of coordinates of PTS for these four lncRNA is given as Additional file 3.

#### Correlation analysis using gene expression data for Refseq and lncRNA genes

We explored the correlation between lncRNAs and the Refseq genes with which these lncRNAs were shown to form a triplex structure at their promoter region. Hence, we derived a list of such triplex forming lncRNAs and their respective genes as predicted in our analysis. There were in total 343 lncRNA transcripts and 17,341 Refseq genes. We performed a correlation analysis using gene expression data (in terms of FPKMS; in-house data of 2179 cell lines not included in this manuscript) for these lncRNAs and Refseq genes. With Pearson’s algorithm in R package; rcorr function was used to find the positively and negatively correlated lncRNA and Refseq genes. The transcripts with an empirical r-score greater than 0.80 were considered to be positively regulating while transcripts with r-score less than −0.80 were considered to be negatively regulating and p value less than 0.01 was considered significant and selected. Using Cytoscape v3.4.0 [31], we constructed a correlation network for lncRNAs and Refseq genes having correlation value greater than 0.80.

### Experimental validation

#### Selection of triplex lncRNA and target duplex site

In biological conditions the presence of R-motif i.e. when the triplex forming strand is purine rich is more biologically relevant than the Y-motif (pyrimidine motif) because Y motif requires the cytosine of the TFO (Triplex Forming Oligonucleotide) to be in protonated form thus requiring an acidic pH which is usually not the optimum pH for biological system. Moreover, the computational predictions showed putative triplex site were enriched in the promoter region when compared to the other genomic features of the gene. Therefore for biophysical validation we focused on these R motif forming triplex lncRNA that could potentially interact with gene promoter regions. One of the lncRNA RP11-84A19 (ENST00000453837) was chosen and among the few predicted gene targets we chose three of the genes for validation i.e. KIAA1324, PROX1, BCL9. At each gene target promoter the lncRNA was able to form more than one triple helix combination (Additional file 4). Based on the number of mis-matches and the length of the PTS, only one combination for each gene was selected for biophysical validation (Table 1). Hence for further experiments the DNA triplex motif and lncRNA triplex motif was used as DNA oligo and RNA TFO respectively.

**Table 1:**
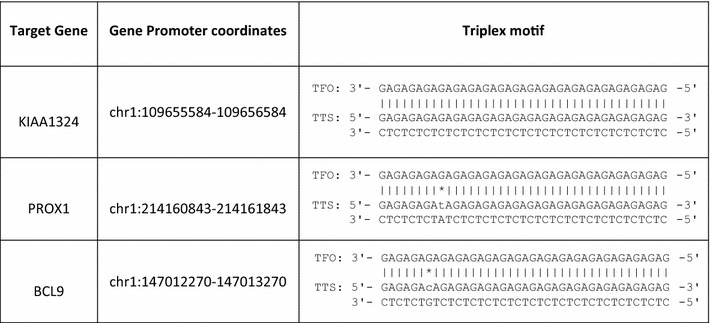
Selected gene targets (DNA duplex or TTS) for biophysical validation experiments of RP11-84A19 lncRNA (RNA TFO)

#### Gel retardation assay

The two DNA oligos that form the duplex were allowed to form the duplex by annealing them in a 1:1 ratio of 1 µM each. Then the third strand i.e. the RNA TFO was added in increasing concentrations from a ratio of 1:1–1:6 (1–6 µM) and allowed to form the triplex in the triplex forming buffer (10 mM Tris pH 7.5, 25 mM NaCl and 10 mM MgCl_2_). The mix of oligos was incubated at 25 °C for 2 h. The formed complexes were analysed on a 10% Native page gel stained with EtBr dye.

#### Thermal UV melting experiments

UV melting assays were performed in Cary 100 varian spectrophotometer equipped with a thermoelectrically controlled cell holder. Triplexes were again formed by duplex annealing followed by addition of RNA TFO in a triplex forming buffer (10 mM Tris pH 7.5, 25 mM NaCl and 10 mM MgCl_2_). The 1:3 ratio was selected for this assay wherein 1 µm was the duplex and 3 µm RNA TFO was added to form effective 1 µm triple helical structure. Both triplex and duplex were subjected to denaturation in a temperature dependent cycle from 90 to 15 °C with a ramp rate of 0.5 °C/min and the absorbance was measured at 260 nm. The data points were also collected at 0.5 °C interval for each sample. The melt curves were plotted with normalized absorbance.

#### Circular dichroism studies

Nucleic acid secondary structure detection was done using Jasco circular dichroism spectrophotometer. The measurements were taken from 300 to 200 nm wavelength in a 1 cm path length quartz cuvette with a wavelength step of 1 nm. Three successive scans were measured for each sample at 25 °C and then plotted as an average single curve. The sample was 2 µm duplex and 6 µm RNA TFO (1:3 ratio) that formed an effective 2 µm triple helical structure in triplex forming buffer (10 mM Tris pH 7.5, 25 mM NaCl and 10 mM MgCl_2_).

## Results

### Genome wide distribution triplex forming lncRNAs

**Fig. 1:**
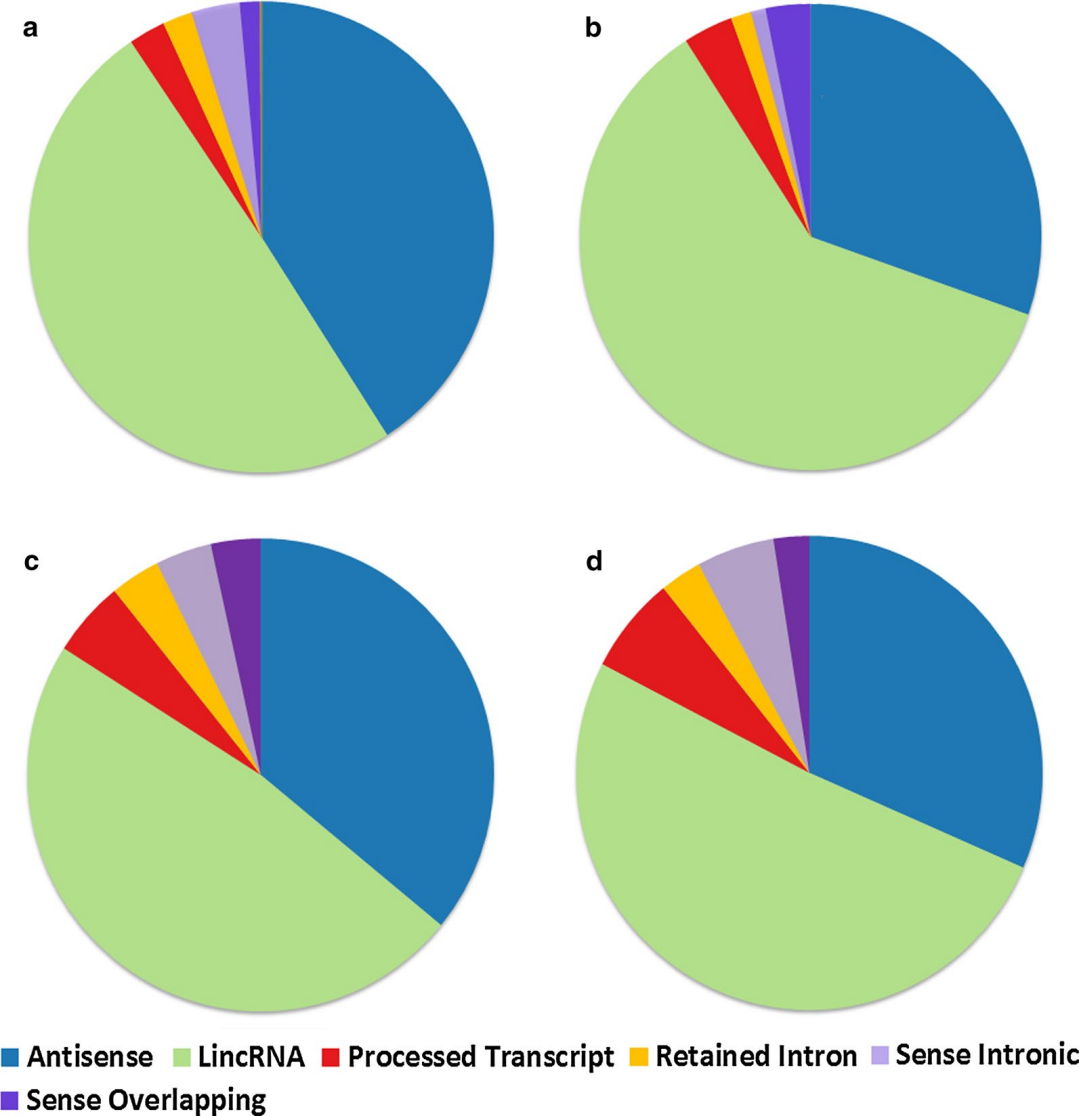
Proportion of lncRNAs acting as potential third strand of PTS across GENCODE biotypes. **a** Distribution of 23,898 GENCODE v19 lncRNAs across six subclasses. **b** Distribution of 286 lncRNAs forming purine motif **c** 231 lncRNAs forming pyrimidine motif and **d** 237 lncRNAs forming mixed motif across the six classes including Antisense, LincRNA, Processed Transcript, Retained Intron, Sense intronic and Sense Overlapping

Prediction of potential triplex forming regions in dsDNA using a brute force approach implemented in Triplexator predicted a total of 6,195,448 forming Purine motif (R), 6,329,571 forming Pyrimidine motif (Y) and 16,652,853 forming Purine–Pyrimidine motif (or mixed/M) potential triplex sites (PTS) in the human genome. The numbers of lncRNAs acting as potential third strand (RNA TFO) in a dsDNA: RNA triplex structure and forming R, Y and M motifs at these PTS sites are 286, 231 and 237 respectively. The complete statistics for PTS sites and their respective motif sequences is given in Table 2 and list of predicted PTS sites with respective target coordinates is given in Additional files 5, 6 and 7. The overlap between the numbers of lncRNA transcripts forming the three motifs has been shown in the venn diagram (Additional file 8). Majority of lncRNAs from GENCODE database as well as the three motif categories were falling in lincRNA and antisense subclasses as shown in Fig. 1a–d. But when we compare the proportion of lncRNAs from three motifs to GENCODE we observed enrichment for lincRNAs, sense overlapping and processed transcript subclass while antisense and sense intronic transcripts showed depletion. The distribution of the annotation of the PTS lncRNAs (B, C and D) and GENCODE lncRNAs (A) in different sub-classes is detailed in Fig. 1.

**Table 2:**
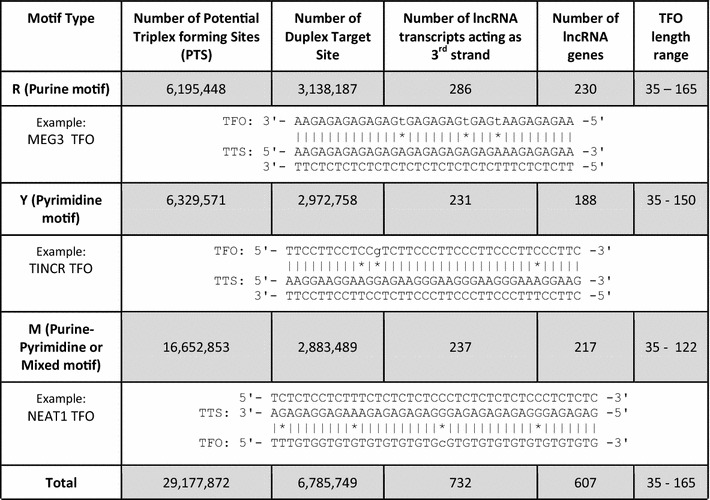
Statistics of the PTS sites in our study

*TFO* triplex-forming oligos (lncRNA sequence), *TTS* triplex target sites (Duplex DNA sequence)

| The complementary base pair

* Non-complementary base pair

**Fig. 2:**
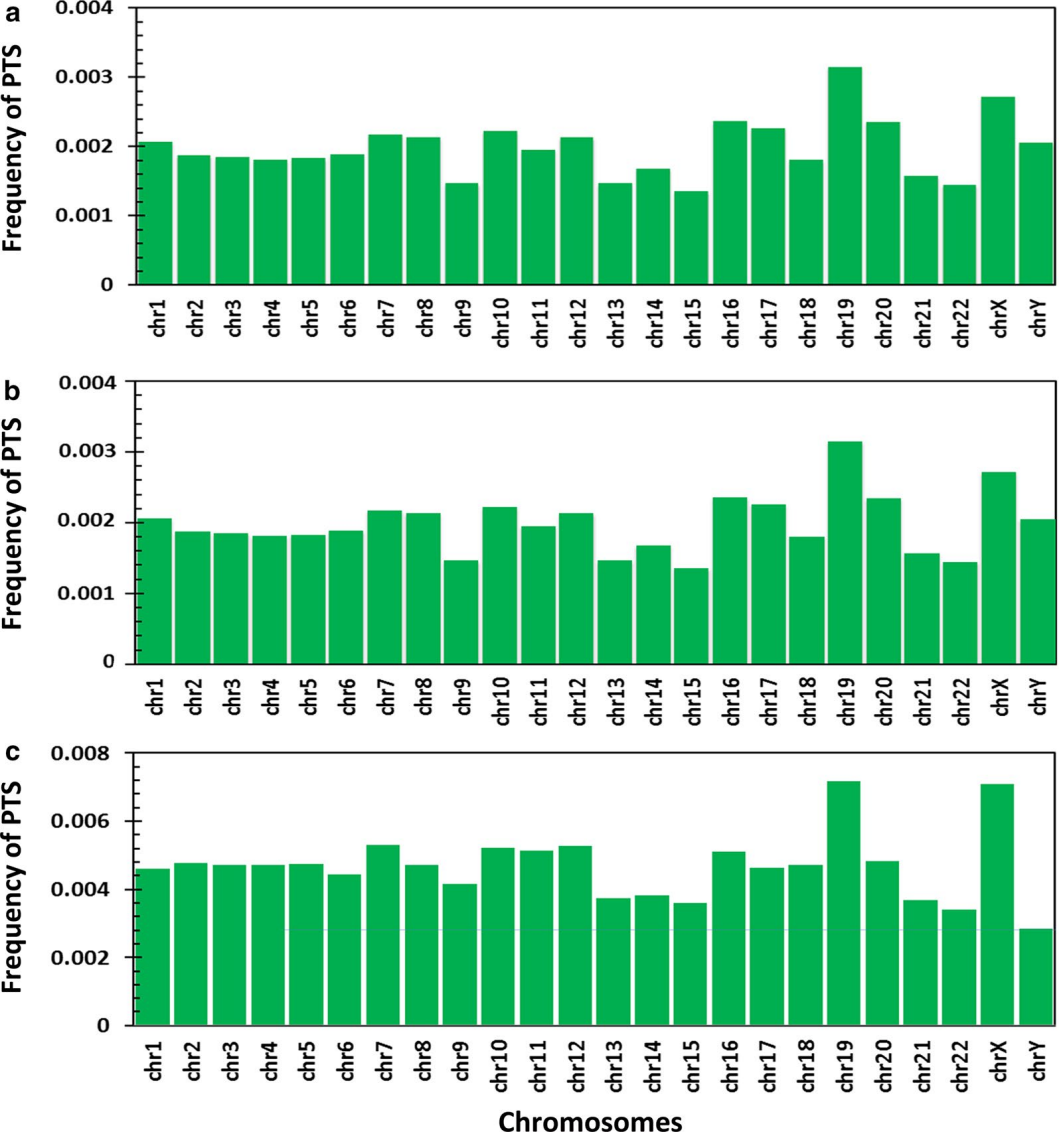
Distribution of PTS frequency across human chromosomes. **a** Mapping frequency of potential triplex sites (PTS) across forming purine motif **b** (PTS) across forming pyrimidine motif **c** (PTS) across forming mixed motif across Human chromosomes

We compiled the mappings of PTS across all the Human chromosomes. Analysis for R and Y motifs revealed a mean mapping frequency of 2 × 10^−3^ per base. Chromosome 19 had a highest mapping frequency of 0.003 per base and Chromosome 15 had least mapping frequency of 1 × 10^−3^ per base. While mean mapping frequency for M motif was 5 × 10^−3^, Chromosome 19 having mapping frequency of 7 × 10^−3^ per base (423,868 PTS) and Chromosome Y having mapping frequency of 3 × 10^−3^ (168,402 PTS) (Fig. 2). An independent evaluation of 100,000 random regions in the genome revealed an average PTS frequency of 2.6 × 10^−5^ per base (Additional file 9).

### Distribution of potential triplex forming sequence stretches in genomic features associated with RefSeq genes

**Fig. 3:**
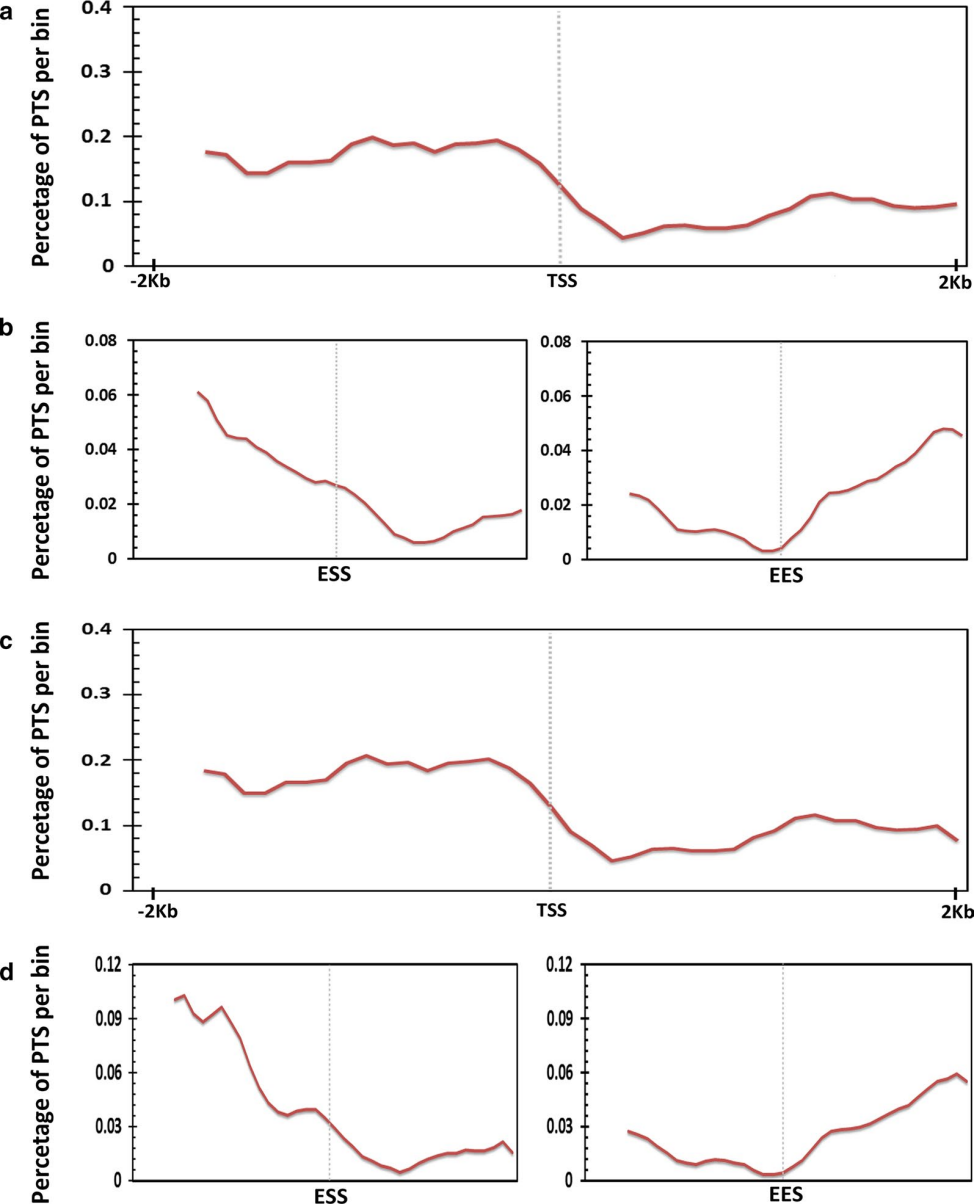
Distribution of purine motif forming PTS across the transcription start sites (TSS). **a** and across the Exon–Intron–Exon boundaries (*ESS* exon start site and *EES* exon end site) **b** of RefSeq genes and genomic features associated with RefSeq genes. Distribution of pyrimidine motif forming PTS across TSS (**c**) and across ESS and EES (**d**)

**Fig. 4:**
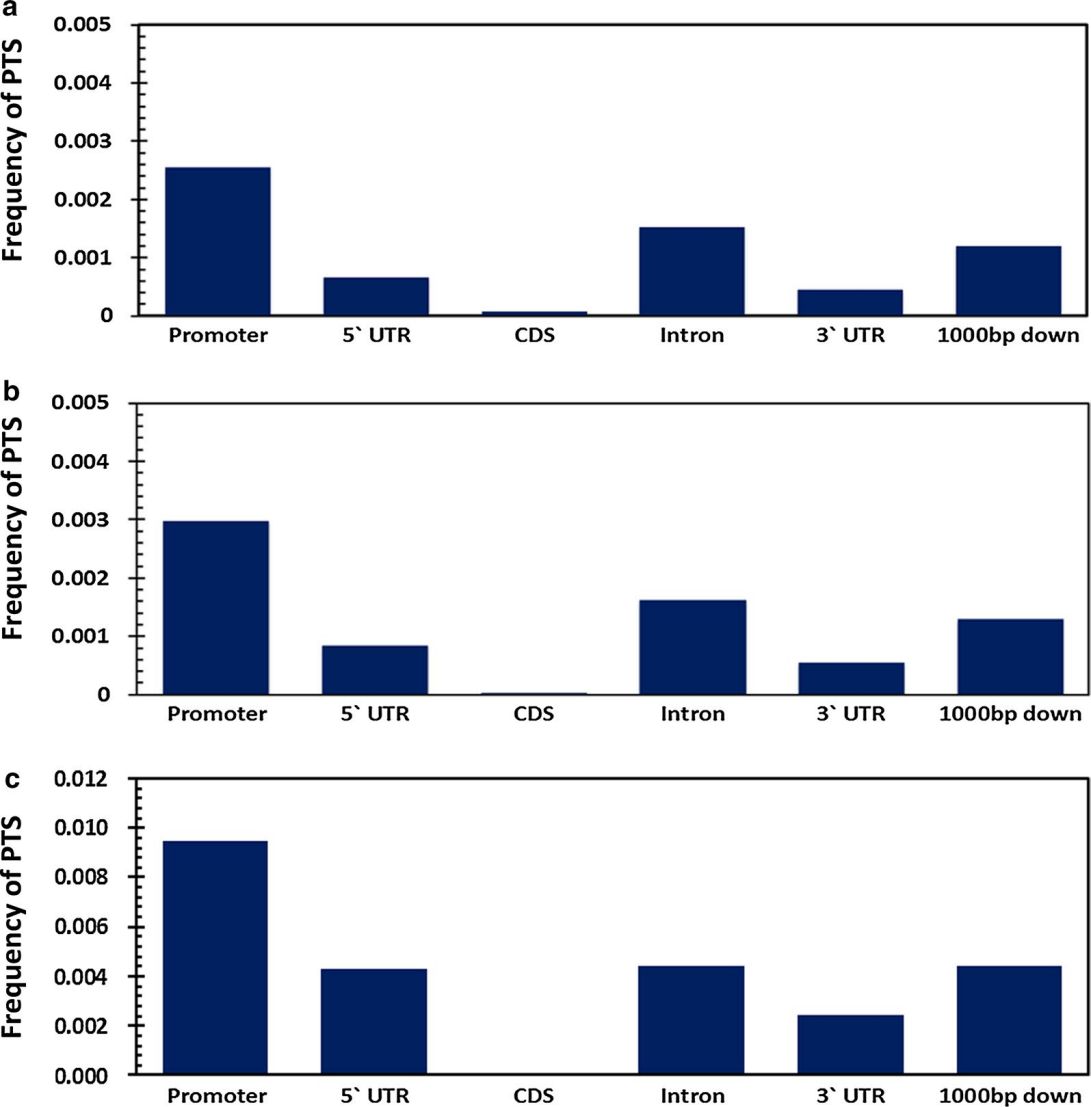
**a** Distribution of purine motif PTS across **b** pyrimidine motif of RefSeq genes and genomic features associated with RefSeq genes **c** mixed motif. The frequency of PTS was plotted for five distinct genomic features associated with the genes including 1000 bases upstream of TSS (promoter), 5′ Untranslated region (5′UTR), Protein-coding exon (CDS), Intron, 3′ Untranslated region (3′UTR) and 1000 bases downstream of transcription end site (1000 bp down)

We initially analysed the frequency of distribution of PTS across the promoters of Refseq genes. Analysis revealed an association of PTS and the core promoter region of genes. The density of PTS across the gene was found attenuated from TSS to TES. A total of 717 and 709 genes were found to have PTS sites forming R and Y motif respectively in the core promoter. No general positional preference was observed in the gene body. We also could not find any enrichment for specific functional classes as annotated by Gene Ontology for the genes which had promoter PTS. The pattern of mapping of PTS with respect to the gene body is summarised in Fig. 3. Further distribution of the potential DNA:DNA:RNA triplex across the RefSeq genes were computed across five major features, namely the 5′UTR, CDS, 3′UTR, Introns, 1000 base upstream of TSS (Promoter) and 1000 bases downstream of the transcription end-site (Fig. 4). The counts were normalised by million bases in each feature analysed. Out of the total of 20,04,034 PTS mapped, a total of 8354 mapped to the 5′UTRs while 2693 mapped to the CDS and 15,301 mapped to the 3′UTR of RefSeq genes. In addition, a total of 18,68,744 PTS mapped to the introns. Analysis of the distribution revealed stark differences in the distribution of the sites. The sites were largely found enriched in the promoters and in the introns compared to the chromosomal average. A paucity of triplex sites was evident across the CDS and in the 3′ and 5′ UTRs. The promoter density of potential triplex sites (PTS) was almost double than that of the introns. Similar trend was also observed for PTS forming pyrimidine and purine–pyrimidine motifs. The frequency distribution for all the three types of motif is summarised in Fig. 4.

### Biophysical validation of triplex structure

**Fig. 5:**
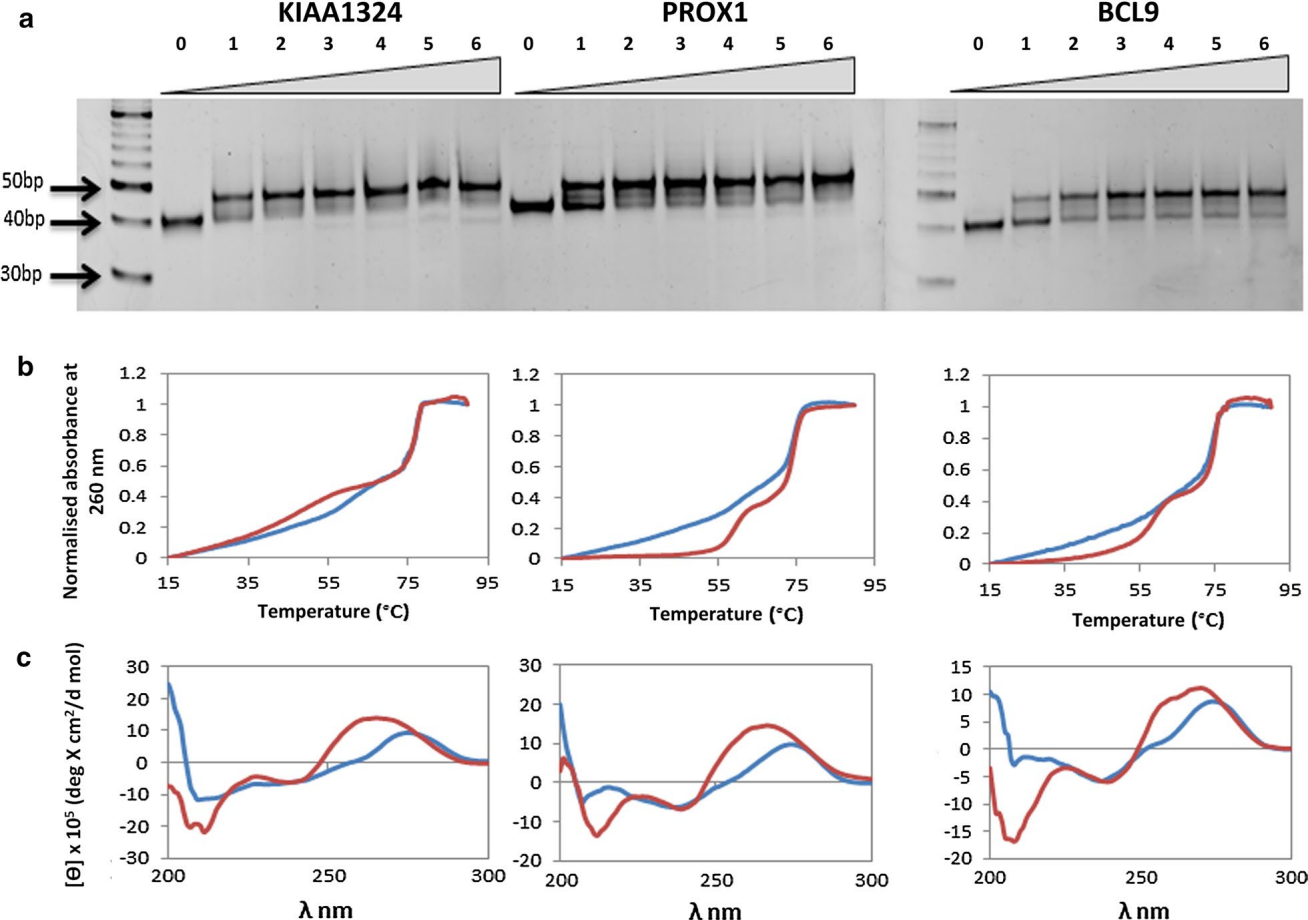
**a** Gel Retardation Assay: duplex DNA incubated alone (*lane 1*) or with increasing concentrations of RNA TFO depicting gel mobility shift with formation of triplex. **b** UV melting assay: UV spectra of duplex and triplex (1:3::duplex:RNA TFO) representing the biphasic melting transitions for triplex (*red*) and single melting peak for duplex (*blue curve*). **c** CD spectroscopy: CD spectra of duplex and triplex (1:3::duplex:RNA TFO) represented with *blue* and *red* curves respectively, indicating the sharp negative peak obtained at 210 nm for the triplex

The lncRNA and three of its gene target promoter region were validated for forming potential triplex structure using biophysical based methods. In Gel retardation assay, triplexes should move slower than its duplex counterpart, similarly we could also observe the shift in gel mobility with increasing concentrations of the RNA TFO for all the three genes (Fig. 5). For *KIAA1324* and *PROX1* most of the duplex had shifted into triple helical structure at 1:2 ratio (DNA duplex:RNA TFO) and for the *BCL9* gene at 1:3 concentration the maximum triplex could be observed which saturated for further concentrations. UV melting absorbance spectroscopy also provides evidence for triple helical structures wherein biphasic melting transitions are observed for triplexes in contrast to a single melting peak obtained for a duplex structure. The first melting temperature i.e. Tm in case of triplexes correspond to the third strand denaturation that was bonded through reverse hoogsteen bonds which are weaker than watson–crick base pairing and thus the lower Tm. While the second Tm is the denaturation of the duplex i.e. the watson–crick base pairing denaturation and hence the higher Tm was obtained. We could demonstrate the presence of two melting temperature in all the three triple helical structures and a single Tm for the counterpart duplexes (Fig. 5). Apart from UV melting we also employed CD spectroscopy to detect nucleic acid secondary structures where triplexes are known to show a signature negative peak at 210 nm [32–34]. Similar negative peak was detected in all the triplexes for all three gene targets (Fig. 5).

### Paucity of potential triplex forming sequence stretches across the promoters of tissue-specific genes in comparison to housekeeping genes

We could explore whether promoters of genes and their functional classes are tissue specifically co-related with PTS sites. Additionally, we evaluated the frequencies of PTS across two distinct classes of genes: Housekeeping genes and tissue specific genes. The datasets for housekeeping genes included 2064 housekeeping genes and 2293 tissue specific genes identified by a recent meta-analysis of microarray datasets [30]. Our analysis revealed that there was a selective depletion of PTS across promoters of tissue specific genes compared to that of housekeeping genes (Chi square 932054.574 and p-value 0) (Additional file 10).

### Association of potential triplex forming sequence stretches with CpG islands, transcription factor binding sites and enhancers

**Fig. 6:**
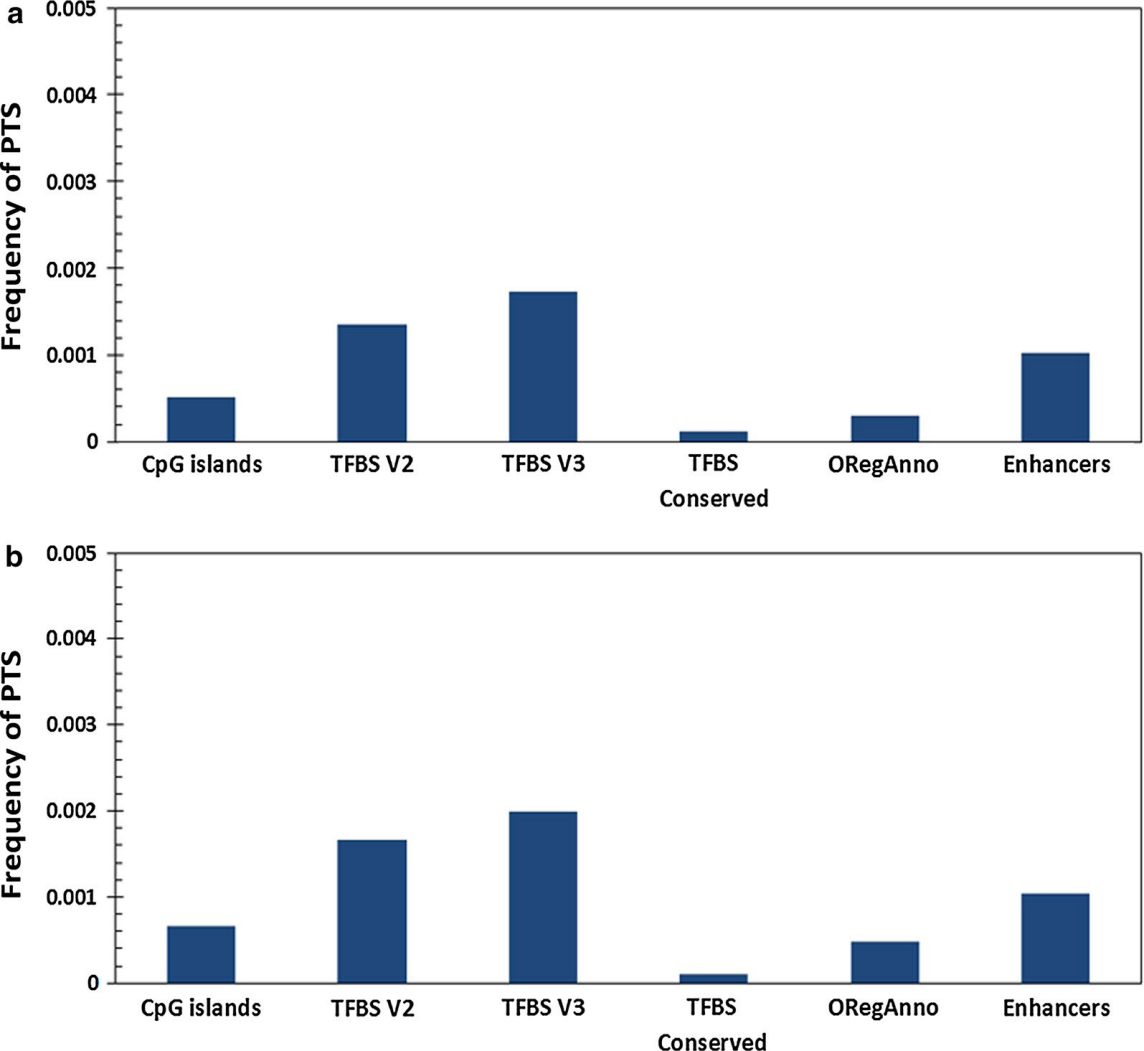
Frequency of **a** purine motif forming PTS and **b** pyrimidine motifs PTS across regulatory regions including CpG islands, sites of Transcription Factor binding Clustered V2 (TFBS), Transcription Factor Binding Clustered V3 (TFBS V3), Conserved Transcription Factor binding sites (TFBS Conserved), Literature Curated regulatory regions (Open Regulatory Annotations: ORegAnno) and Enhancers

We additionally explored the association of PTS with core promoter elements. CpG islands have been one of the well-studied promoter element associated with regulatory roles. CpG islands are largely located in the core promoter of genes, and it has been estimated that approximately 70% of Human genes have CpG islands in the proximal promoter and has been extensively studied with respect to their regulatory roles. We considered a total of 28,691 CpG island sites from the UCSC Genome Browser and compared the frequency of PTS sites in association with CpG islands. Analysis revealed that PTS are depleted at CpG islands, suggesting core regulatory elements delineated by CpG islands and largely not regulated through PTS. We further evaluated a number of other regulatory features around the TSS of genes, including transcription factor binding sites, enhancers and regulatory elements annotated by ORegAnno in the genome. A systematic paucity of PTS in all of these regulatory regions was observed, except for Transcription factor binding sites. This prompted us to closely evaluate the association of PTS with specific transcription factor binding sites in the human genome. Frequency for PTS for these regulatory elements is summarized in Fig. 6.

### Distribution of potential triplex forming sequence stretches across transcription factor binding sites

The availability of experimental datasets for transcription factor binding sites using ChIP-seq from the ENCODE consortium provides an opportunity to analyse the binding sites for transcription factors which overlap with PTS. This could provide insights into the regulatory networks modulated through lncRNA mediated triplex formation in regulating genes. We analysed a total of 692 HAIB (Myers—Hudson Alpha Institute for Biotechnology lab), 55 UTA (UT Austin lab) and 359 SYDH (Snyder—Stanford University) ENCODE datasets. The associations of transcription factor binding sites were independently evaluated for each of the transcription factor ChIP-seq datasets for each of the cell line. We considered a transcription factor binding site to be enriched only if majority of the cell line datasets showed a significant enrichment or association. Analysis revealed a total of 25 transcription factors, showing significant association with PTS in the genome in majority of the cell lines analyzed.

Out of the ChiP-seq datasets, significant enrichment to PTS was observed only for transcription factors CTCF and NSRF. CTCF is known to play an important role in mediating inter and intra chromosomal interactions [35–37] and being a transcription factor it has higher binding frequency at the gene promoter regions. The enrichment of the transcription factor was tested (Additional file 11). Additional file 12 shows the distribution of five transcription factors across the 2 kb upstream and downstream of transcription start site (TSS). Since CTCF and NRSF binding site were enriched for the PTS, we hypothesized that PTS would contribute to the 3D chromatin organization in the genome, or both the proteins formed major player in chromatin organisation [38, 39]. Towards this end, we analysed publically available Hi-C datasets.

### Hi-C interaction domains enriched for potential triplex forming sequence stretches sites

To understanding the distribution of the PTS sites at genome-wide scale with a higher resolution we used the Hi-C interaction domains datasets available for four cell-lines from http://egg.wustl.edu/d/hg19/. The derived processed data in form of peaks was exactly overlapped on the PTS sites corresponding to the three types of motifs (R, Y & M). The complete list for possible Hi-C interaction domains with PTS sites is given as Additional files 13, 14, 15, 16, 17, 18, 19, 20, 21. We next analysed enrichment or depletion of the hi-C interactions sites using statistical test. In our analysis, we observed a significantly higher frequency for Hi-C domains at the PTS sites. The results of statistical test with their respective p-value for the three motif is summarized in Additional file 22.

### Distribution of potential triplex forming sequence stretches with histone modifications

**Fig. 7:**
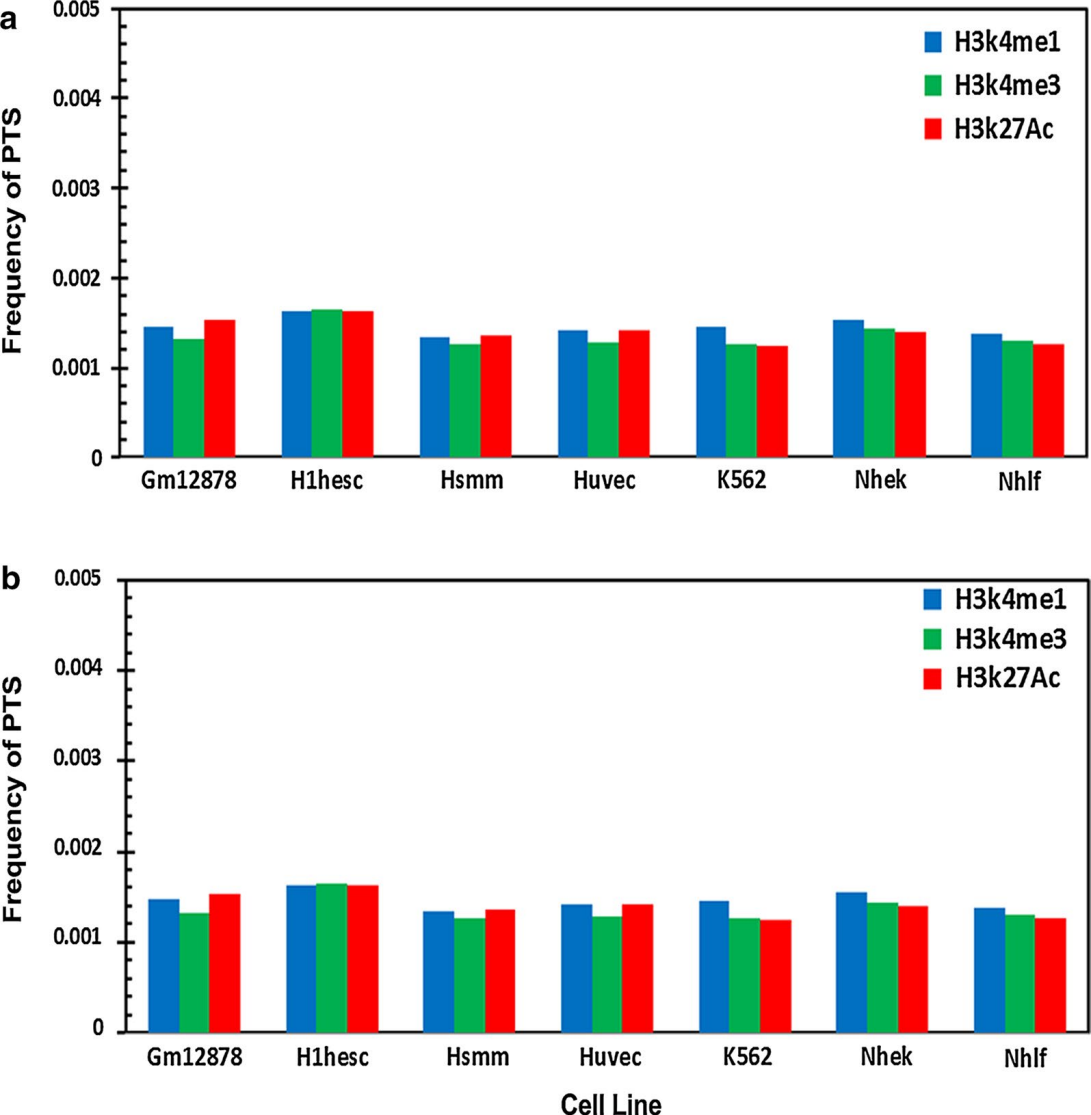
Comparative analysis of the association of **a** purine motif forming PTS and **b** pyrimidine motifs forming with different histone modification marks H3K4Me1, H3K4me3 and H3K27Ac

Apart from CpG islands, the availability of experimental datasets for histone modifications using genome-wide approaches has been made available in recent years. We used three histone modification datasets available in the Regulation track of UCSC genome browser, namely H3K4Me1, H3K4me3 and H3K27Ac. Seven independent cell lines in which the histone modifier loci were assayed, were considered for the analysis. A comparative analysis of the association of PTS with each of the three datasets is summarized in Fig. 7. None of the specific histone marks analysed in the present study showed enrichment at PTS compared to the chromosomal average. In fact most of the histone modifier sites showed depletion at PTS suggesting that PTS are generally devoid of modified histones. In addition, we also mapped PTS to the 98 DnaseI hypersensitivity datasets from ENCODE and observed that there was a dearth of such features at triplex sites (Additional file 23).

### Association of potential triplex forming sequence stretches with repeat elements

**Fig. 8:**
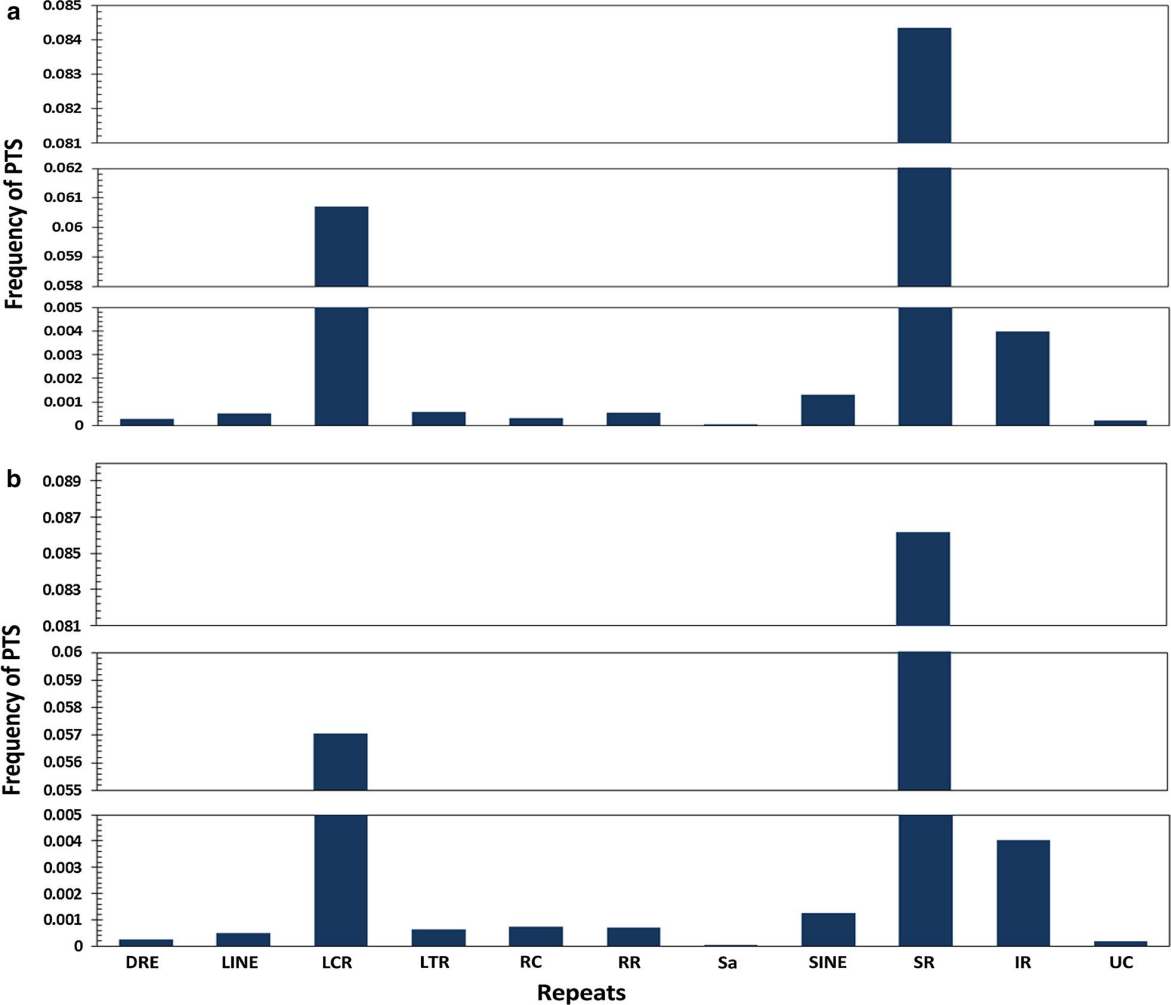
Distribution of **a** purine motif forming PTS and **b** pyrimidine motifs forming PTS across the ten different classes of repeats which includes DNA repeat elements (DRE), long interspersed nuclear elements (LINE), low complexity repeats (LCR), long terminal repeat elements (LTR), rolling circle (RC), RNA Repeats (RR), Satellite (Sa), short interspersed nuclear elements (SINE), simple repeats (SR), interrupted repeats (IR) and unknown (UC)

We additionally analysed the association of PTS with respect to simple and interrupted repeats in the genome. Analysis suggested PTS as largely associated with repeats, having huge predilection for association with simple repeats compared to interrupted repeats. The distribution of PTS in the selected gene features are summarized in Fig. 8. Apart from the simple and interrupted repeats, we also analyzed the association of PTS with other major repeat classes.

### Co-expression networks for positively correlating lncRNAs

We analysed the correlation of expression (in terms of FPKM; data not included in this study) of lncRNAs and their cognate targets by virtue of the lncRNA binding to the promoter of the target gene. Of the entire set of 343 lncRNAs targeting 17,341 Refseq genes only 23 lncRNAs showed a positive correlate 51 Refseq genes (r ≥ 0.8 and p ≤ 0.01), while none showed significant negative correlation. The same is depicted as network in Additional file 24.

## Discussion

Current understanding of lncRNA function is limited to a few candidate lncRNAs when compared to the large number of lncRNAs annotated till date [40]. Increasing evidence suggests that at sequence levels lncRNAs play critical roles, although they have low sequence conservation, the promoter region are largely conserved with respect to their exons as in case of mRNA promoters [41, 42]. The present functional associations of lncRNAs are largely limited to ‘guilt by association’ methods. The integration of omics datasets can help us predict the potential functional role of lncRNAs. For example, diverse number of lncRNAs have been closely associated with p53 and found to be regulated by p53 in turn being responsible for maintaining cellular stabilities [43]. Computational approaches would provide valuable insights in understanding the biogenesis, regulation and function of lncRNAs thus could provide a huge impetus in the field. With the availability of sequencing based approaches to understand bio-molecular interactions—be it Protein: RNA, RNA: RNA or RNA: DNA interactions are also described previously in this manuscript.

Previous studies have identified limited lncRNAs such as MEG3, DHFR, FENDRR, HOTAIR which can participate in DNA duplex—lncRNA triplex formation. We employed a computational approach to screen the human genome for possible triple helical formation mediated through lncRNAs. We screened for 23,898 transcripts annotated as lncRNAs in the GENCODE annotation (v19) across the human genome (hg19) for potential triplex forming sequence stretches (PTS). The calculated PTS frequencies were compared across five major features, namely 5′UTR, CDS, 3′UTR, Introns, Promoter and 1000 bases downstream of the transcription termination sites. Additionally, annotation of these regions was done by mapping of experimental regulatory regions, different classes of repeat regions and transcription factors (TF) derived from UCSC Table Browser. A number of lncRNAs have been shown to interact with transcription factors as evident by their higher frequency in promoter region. To elucidate possible functional roles of triplex forming lncRNA, we counter checked those lncRNAs with the functionally annotated lncRNAs from lncRNAdb database. Out of the total 184 lncRNAs from lncRNAdb database (last accessed in April 2017) [44] only nine showed to have triplex forming capability, namely; MEG3 (ENST00000453837.1)—R motif, HOTAIRM1 (ENST00000434063.3)—R motif, ATXN8OS (ENST00000414504.2)—R motif, BCYRN1 (ENST00000418539.1)—R motif, LINC00599 (ENST00000521242.1)—Y motif, OTX2-AS1 (ENST00000534909.2)—Y motif, TINCR (ENST00000448587.1)—Y motif, SNHG16 (ENST00000448136.1)—M motif, NEAT1 (ENST00000501122.2)—M motif, However, detailed functional analyses of many such genes are needed in order to derive at a clearer picture of the roles of lncRNAs. The motif sequence for the previously known and unknown triplex forming lncRNA categorized into the specific type of motif they form is given in Table 3.

**Table 3:** Classification of previously known and unknown lncRNA into three types of motifs

Type of motif	Previously known (K) lncRNAs forming triplex structure		Previously unknown [UK] and known [K] (predicted from our study)	
	lncRNA name	Number of TFO	lncRNA name	Number of TFO
R	MEG3 [20]	10	MEG3 [K]	3
	HOTAIR [45]	3	HOTAIRM1 [UK]	6
	FENDRR [16]	~1	ATXN8OS [UK]	10
			BCYRN1 [UK]	6
Y	DHFR [17]	~1	TINCR [UK]	32
			OTX2-AS1 [UK]	2825
			LINC00599 [UK]	37
M	–	–	NEAT1 [UK]	596
			SNHG16 [UK]	204
			SCARNA9 [UK]	63
			FMR1-AS1 [UK]	118

Computationally, we found promoter region to have more putative triplex sites and we even proceeded to validate few of the promoter interacting lncRNA for its triplex forming capability at target gene promoters through biophysical techniques. The target genes for example KIAA1324 has been found into correlate with survival in certain carcinomas [46] and may be important for cellular response to stress [47], indicating regulation of such genes in a cell by triplex structure at the promoter region through an lncRNA which could reveal functional role of an novel lncRNA and could be valuable source of information.

Our analysis suggests significant enrichment of PTS sites with specific DNA binding proteins, specifically NRSF and CTCF. Incidentally these proteins are also key components which participate in chromatin organization and regulation [38, 39]. The transcriptional repressive protein, NRSF/REST (repressor-element-1-silencing transcription factor) is responsible for the inhibition of expression of neuron-specific genes. Guardavaccaro and group proposed that degradation of this protein in G2 phase of cell cycle is necessary to depress genes involved in mitosis. They have shown degradation of REST by ubiquitin ligase SCF β-TrCP in G2 phase allowing transcriptional depression of Mad2, an essential component of the spindle assembly checkpoint. CTCF (CCCTC-binding factor) has been previously shown to be involved in regulation of transcriptional by binding to chromatin insulators, hence preventing the direct interaction of promoter and enhancers/silencers. But recently Ong and Corces highlighted involvement of CTCF in framing boundaries across topologically associating domains in chromosomes facilitating CTCF to interact between transcription regulatory sequences. Our independent analysis of PTS sites at interaction domains suggest significant enrichment, suggesting one of the major roles of PTS forming lncRNAs could be in chromatin organisation by closely binding to CTCF/NRSF proteins.

Further studies will be necessary to completely elucidate and validate the functional interactions as predicted in our analysis. With advanced high-throughput approaches secondary structure, protein-binding motifs and other features in the primary sequence could be determined in detail, to present a global landscape of elements in lncRNAs.

## Conclusions

Our study focuses on computational identification of potential triplex forming sites mediated through lncRNAs. In total, we screened 23,898 lncRNA transcripts for their PTS frequencies across five major genic features, 5′UTR, CDS, 3′UTR, Introns, Promoter and 1000 bases downstream of the transcription termination sites, showing enrichment in promoter and intronic regions. As computational analysis revealed enrichment for PTS within the gene promoter regions, henceforth we successfully validated presence of triple helical structure formed by a lncRNA with its three target gene promoter region through biophysical methods including gel retardation, UV absorbance and CD spectroscopy. In addition, we observed enrichment of CTCF and NRSF in PTS sites, these proteins are known to play crucial roles in chromatin organisation, hence we hypothesized that PTS could be playing an important role in contributing to 3D chromatin organisation and its regulation mediated through these proteins. Our present study encompasses a genome-wide distribution of PTS sites across human genome mediated through lncRNAs and their possible functional roles.

## Additional files

**Additional file 1.** The table details the parameters used for running triplexator tool.

**Additional file 2.** The list of four lncRNAs previously published in literature and forms triplex structure. The potential interactions were predicted using different parameters of triplexator tool. The percentage of PTS sites of each of the four lncRNA forming any of the three types of motif, as predicted in our analysis is mentioned in table.

**Additional file 3.** The complete list of coordinates of PTS for the four lncRNA viz. MALAT1, HOTAIR and DHFR.

**Additional file 4.** List of all potential triplex interactions between lncRNA (RP11-84A19.2) and promoter regions of three genes (KIAA1324, PROX1, BCL9).

**Additional file 5.** List of predicted lncRNAs forming Purine motif (R) motif with the human reference genome. The file consists of lncRNA transcript id, lncRNA gene name, lncRNA length, TFO start, TFO stop, chromosome number of TTS, TTS start, TTS stop, motif type, strand orientation and guanine-rate.

**Additional file 6.** List of predicted lncRNAs forming Pyrimidine motif (Y) with the human reference genome. The file consists of lncRNA transcript id, lncRNA gene name, lncRNA length, TFO start, TFO stop, chromosome number of TTS, TTS start, TTS stop, motif type, strand orientation and guanine-rate.

**Additional file 7.** List of predicted lncRNAs forming Purine-Pyrimidine motif (M) motif with the human reference genome. The file consists of lncRNA transcript id, lncRNA gene name, lncRNA length, TFO start, TFO stop, chromosome number of TTS, TTS start, TTS stop, motif type, strand orientation and guanine-rate.

**Additional file 8.** Venn diagram representing the overlap of lncRNAs forming the three types of motifs (R, Y & M).

**Additional file 9.** Distribution of random genomic loci across the Refseq genes and associated genomic features including Promoter, 5′ UTR, CDS, Intron, 3′ UTR and 1000 bases downstream.

**Additional file 10.** Conservation of Housekeeping and Tissue specific genes across the PTS, tested by Chi-square test.

**Additional file 11.** The results of Chi-square test for testing mapping significance of the three transcription factors mapping to PTS. [A] Chi-square test to check the mapping significance of UTA Transcription Factor Binding sites (UTA TFBS) with Potential Triplex Sites. [B] Chi-square test to check the mapping significance of HAIB Transcription Factor Binding sites (HAIB TFBS) with Potential Triplex Sites. [C] Chi-square test to check the mapping significance of SYDH Transcription Factor Binding sites (SYDH TFBS) with Potential Triplex Sites.

**Additional file 12.** Distribution of Transcription factors across the Transcription Start Site (TSS) (A) Pol24h8 (B) NRSF (C) CTCF (D) CMYS (E) CTCFB.

**Additional file 13.** Complete list for possible Hi-C (CCHiC-HeLaS3-G1mid-R2) interaction domains with PTS sites.

**Additional file 14.** Complete list for possible Hi-C (CCHiC-HeLaS3-M-R1) interaction domains with PTS sites.

**Additional file 15.** Complete list for possible Hi-C (CCHiC-HeLaS3-M-R2) interaction domains with PTS sites.

**Additional file 16.** Complete list for possible Hi-C (Hi-C_PTS_CCHiC-HeLaS3CCL2p2-G1-0.25F) interaction domains with PTS sites.

**Additional file 17.** Complete list for possible Hi-C (CCHiC-HeLaS3CCL2p2-G1-1FA) interaction domains with PTS sites.

**Additional file 18.** Complete list for possible Hi-C (CCHiC-HeLaS3CCL2p2-M-0.25FA) interaction domains with PTS sites.

**Additional file 19.** Complete list for possible Hi-C (CCHiC-HeLaS3CCL2p2-M-1FA) interaction domains with PTS sites.

**Additional file 20.** Complete list for possible Hi-C (CCHiC-HeLaS3CCL2p2-M-98percent) interaction domains with PTS sites.

**Additional file 21.** Complete list for possible Hi-C (CCHiC-K562-M-R1) interaction domains with PTS sites.

**Additional file 22.** The results of Chi-square test for testing mapping significance of the Hi-C mapping to PTS. [A] Chi-square test to check the mapping significance of Hi-C interaction domain with Potential Triplex Sites forming purine motif (R). [B] Chi-square test to check the mapping significance of Hi-C interaction domain with Potential Triplex Sites forming pyrimidine motif (Y). [C] Chi-square test to check the mapping significance of Hi-C interaction domain with Potential Triplex Sites forming purine-pyrimidine motif (M).

**Additional file 23.** Distribution of (A) Purine motif forming PTS and (B) Pyrimidine motif forming PTS across 98 DnaseI hypersensitivity datasets from ENCODE.

**Additional file 24.** Correlation network constructed using Cytoscape for 23 lncRNAs and 51 Refseq genes. The Refseq genes are represented by blue node, lncRNAs by red node and the edge (grey) represents the correlation value. The thickness of the edge represents the value ranging from 0.08 to 0.91, thicker the line higher the correlation. [A] The network for lncRNAs forming triplex structure with promoters of Refseq gene predicted with these parameters (-l 35 -e 10 -g 20 -m R/Y/M -fm 0 -of 1 -fr off). [B] Interaction network for HOTAIR and MALAT1 lncRNAs, which are known to form triplex structure from previous literatures [14] and were predicted in our analysis with these parameters (-l 35/40 -e 20/20 -g 20 -m R/Y/M -fm 0 -of 1 -fr off).

## Abbreviations

PTS: potential triplex forming sequence stretches
UTR: untranslated region
CDS: coding DNA sequence
TF: transcription factors
lncRNAs: long noncoding RNAs
R motif: purine motif
Y motif: pyrimidine motif
M motif: purine–pyrimidine motif
DRE: DNA repeat elements
LINE: long interspersed nuclear elements
LCR: low complexity repeats
LTR: long terminal repeat elements
RC: rolling circle
RR: RNA Repeats
Sa: Satellite
SINE: short interspersed nuclear elements
SR: simple repeats
IR: interrupted repeats
UC: unknown
HOTAIR: Hox antisense intergenic RNA
HOTTIP: HOXA transcript at the distal tip
PRC2: polycomb repressive complex 2
TrxG/MII: Trithorax Group/Myeloid/lymphoid or mixed-lineage Leukemia
DHFR: dihydrofolate reductase
MEG3: Maternally Expressed 3
CTCF: CCCTC-binding factor/11-zinc finger protein
NSRF: neuron-restrictive silencer factor
hnRNP: heterogeneous ribonucleoprotein particle
SPHK1: sphingosine kinase 1
NEAT1: Nuclear Enriched Abundant Transcript 1
TTS: triplex target site
PTS: potential triplex site
CHIRP: chromatin isolation by RNA purification
TSS: transcription start site
TES: transcription end site
µM: micromolar
Hi-C: high-throughput chromosome conformation capture
ChIP: chromatin immune precipitation
CHIRP: chromatin isolation by RNA purification
CHART: capture hybridization analysis of RNA targets
H3K4Me1: monomethylation of the 4th residue (a lysine) from the start (i.e., the N-terminal) of the H3 protein
H3K4me3: Trimethylation of H3 lysine 4
H3K27Ac: acetylation at the 27th lysine residue of the histone H3 protein
