# Supplementary material for: Genome-wide computational analysis of potential long noncoding RNA mediated DNA:DNA:RNA triplexes in the human genome
Source: J Transl Med. 2017 Sep 2;15:186. doi: 10.1186/s12967-017-1282-9 (PMC7670996; doi:10.1186/s12967-017-1282-9)
Supplement: Supplementary file 1 — Additional file 1. The table details the parameters used for running triplexator tool. [file 12967_2017_1282_MOESM1_ESM.pdf]

Parameter	Full Form	Definition	Default value	Value used in our study
-l	--lower-length-bound	The minimum length of a TFO, TTS or triplex target sites (TTS-TFO pair)	15	35
-e	--error-rate	The maximal error-rate in % which is allowed for prediction. A match of a feature R with E errors has error-rate of 100*(E/|R|), whereby |R| is the feature length.	5	10
-g	--min-guanine	The minimum guanine content in triplex features. Value ranges between 0 and 100. The minimum guanine rate controls the ratio of guanines required in the any TTS.	<10%	<20%
-m	--triplex-motifs	Specifies the motifs from the canonical triplex-formation rules to be used when searching for TFOs in the third strand:<br>R - the purine motif that permit guanines (G) and adenines (A)<br>Y - the pyrimidine motif that permit cytosines (C) and thymines (T)<br>M - the mixed motif, purine-pyrimdine, that permit guanines (G) and thymines (T)	R,Y,M	R, Y,M
-fm	--filtering-mode	This discards the non-hits<br>0 = greedy approach<br>1 = q-gram filtering	0	0
-of	--output-format	Type of output format<br>0 = Tab-separated Format + Summary Format<br>1 = Triplexator Format + Summary Format<br>2 = Summary Format only	To be specified	1
-fr	--filter-repeats	Activates the filtering of low complexity regions and repeats in the sequence data. It is deactivated as it greatly increases the memory consumption and runtime of Triplexator. However, many repeat regions comply with triplex-formation rules.	off	off
