# Supplementary material for: Genome-wide computational analysis of potential long noncoding RNA mediated DNA:DNA:RNA triplexes in the human genome
Source: J Transl Med. 2017 Sep 2;15:186. doi: 10.1186/s12967-017-1282-9 (PMC7670996; doi:10.1186/s12967-017-1282-9)
Supplement: Supplementary file 2 — Additional file 2. The list of four lncRNAs previously published in literature and forms triplex structure. The potential interactions were predicted using different parameters of triplexator tool. The percentage of PTS sites of each of the four lncRNA forming any of the three types of motif, as predicted in our analysis is mentioned in table. [file 12967_2017_1282_MOESM2_ESM.pdf]

	Type of Motif	L10E10	L20E10	L30E20	L30E10	L35E20	L40E20
**HOTAIR**	**R**	-	44.55	61.95	33.33	93.14(*)	100(*)
	**Y**	-	0	0.50	0	0(*)	0(*)
	**M**	-	55.45	37.55	66.67	6.86(*)	0(*)
**MALAT1**	**R**	-	18.93	65.59	100	72.77(*)	92.86(*)
	**Y**	-	0.67	0.19	0	0(*)	0(*)
	**M**	-	80.40	34.22	0	27.23(*)	7.14(*)
**FENDRR**	**R**	-	68.91	37.57	0	66.67(*)	0
	**Y**	-	15.96	62.43	0	33.33(*)	0
	**M**	-	15.13	0	0	0(*)	0
**DHFR**	**R**	19.15(*)	0	0	0	0	0
	**Y**	34.78(*)	0	0	0	0	0
	**M**	47.57(*)	0	0	0	0	0

L: length; E: error rate; R:Purine motif; Y:Pyrimidine motif ; M: purine-pyrimidine motif; (*) marked: values at the parameter used in our study.
