# Supplementary material for: Genome-wide computational analysis of potential long noncoding RNA mediated DNA:DNA:RNA triplexes in the human genome
Source: J Transl Med. 2017 Sep 2;15:186. doi: 10.1186/s12967-017-1282-9 (PMC7670996; doi:10.1186/s12967-017-1282-9)
Supplement: Supplementary file 4 — Additional file 4. List of all potential triplex interactions between lncRNA (RP11-84A19.2) and promoter regions of three genes (KIAA1324, PROX1, BCL9). [file 12967_2017_1282_MOESM4_ESM.pdf]

LncRNA of interest: ENST00000453837.1 (RP11-84A19.2)

TFO: **lncRNA** **TTS:** **site on the gene promoter where lncRNA forms triplex**

1. **Gene: KIAA1324**

Promoter region (1kb upstream): chr1:109655584-109656584

Potential Triplex Site (PTS):

chr1 109656055 109656090

```
TFO: 3'- GAGAGAGAGAGAGAGAGAGAGAGAGAGAGAGAGAG -5'
         |||||||||||||||||||||||||||||||||||
TTS: 5'- GAGAGAGAGAGAGAGAGAGAGAGAGAGAGAGAGAG -3'
     3'- CTCTCTCTCTCTCTCTCTCTCTCTCTCTCTCTCTC -5'
```

chr1 109656053 109656090

```
TFO: 3'- GAGAGAGAGAGAGAGAGAGAGAGAGAGAGAGAGAGAG -5'
         |||||||||||||||||||||||||||||||||||||
TTS: 5'- GAGAGAGAGAGAGAGAGAGAGAGAGAGAGAGAGAGAG -3'
     3'- CTCTCTCTCTCTCTCTCTCTCTCTCTCTCTCTCTCTC -5'
```

chr1 109656051 109656090

```
TFO: 3'- GAGAGAGAGAGAGAGAGAGAGAGAGAGAGAGAGAGAGAG -5'
         |||||||||||||||||||||||||||||||||||||||
TTS: 5'- GAGAGAGAGAGAGAGAGAGAGAGAGAGAGAGAGAGAGAG -3'
     3'- CTCTCTCTCTCTCTCTCTCTCTCTCTCTCTCTCTCTCTC -5'
```

chr1 109656049 109656091

```
TFO: 3'- GAGAGAGAGAGAGAGAGAGAGAGAGAGAGAGAGAGAGAGttG -5'
         |||||||||||||||||||||||||||||||||||||||**|
TTS: 5'- GAGAGAGAGAGAGAGAGAGAGAGAGAGAGAGAGAGAGAGAGG -3'
     3'- CTCTCTCTCTCTCTCTCTCTCTCTCTCTCTCTCTCTCTCTCC -5'
```

chr1 109656047 109656086

```
TFO: 3'- GAGAGAGAGAGAGAGAGAGAGAGAGAGAGAGAGAGAGAG -5'
         |||||||||||||||||||||||||||||||||||||||
TTS: 5'- GAGAGAGAGAGAGAGAGAGAGAGAGAGAGAGAGAGAGAG -3'
     3'- CTCTCTCTCTCTCTCTCTCTCTCTCTCTCTCTCTCTCTC -5'
```

chr1 109656045 109656084

```
TFO: 3'- GAGAGAGAGAGAGAGAGAGAGAGAGAGAGAGAGAGAGAG -5'
         |||||||||||||||||||||||||||||||||||||||
TTS: 5'- GAGAGAGAGAGAGAGAGAGAGAGAGAGAGAGAGAGAGAG -3'
     3'- CTCTCTCTCTCTCTCTCTCTCTCTCTCTCTCTCTCTCTC -5'
```

chr1 109656043 109656082

```
TFO: 3'- GAGAGAGAGAGAGAGAGAGAGAGAGAGAGAGAGAGAGAG -5'
         |||||||||||||||||||||||||||||||||||||||
TTS: 5'- GAGAGAGAGAGAGAGAGAGAGAGAGAGAGAGAGAGAGAG -3'
     3'- CTCTCTCTCTCTCTCTCTCTCTCTCTCTCTCTCTCTCTC -5'
```

chr1 109656041 109656080

```
TFO: 3'- GAGAGAGAGAGAGAGAGAGAGAGAGAGAGAGAGAGAGAG -5'
         |||||||||||||||||||||||||||||||||||||||
TTS: 5'- GAGAGAGAGAGAGAGAGAGAGAGAGAGAGAGAGAGAGAG -3'
     3'- CTCTCTCTCTCTCTCTCTCTCTCTCTCTCTCTCTCTCTC -5'
```

chr1 109656037 109656078

```
TFO: 3'- AGGAGAGAGAGAGAGAGAGAGAGAGAGAGAGAGAGAGAGAG -5'
         |*|||||||||||||||||||||||||||||||||||||||
TTS: 5'- AAGAGAGAGAGAGAGAGAGAGAGAGAGAGAGAGAGAGAGAG -3'
     3'- TTCTCTCTCTCTCTCTCTCTCTCTCTCTCTCTCTCTCTCTC -5'
```

```
chr1      109656038           109656076
TFO: 3'- AGAGAGAGAGAGAGAGAGAGAGAGAGAGAGAGAGAGAGAG -5'
         ||||||||||||||||||||||||||||||||||||||
TTS: 5'- AGAGAGAGAGAGAGAGAGAGAGAGAGAGAGAGAGAGAGAG -3'
     3'- TCTCTCTCTCTCTCTCTCTCTCTCTCTCTCTCTCTCTCTC -5'

chr1      109656035           109656074
TFO: 3'- GAGAGAGAGAGAGAGAGAGAGAGAGAGAGAGAGAGAGAG -5'
         ||*||||||||||||||||||||||||||||||||||||
TTS: 5'- GAAAGAGAGAGAGAGAGAGAGAGAGAGAGAGAGAGAGAG -3'
     3'- CTTTCTCTCTCTCTCTCTCTCTCTCTCTCTCTCTCTCTC -5'

chr1      109656033           109656072
TFO: 3'- GAGAGAGAGAGAGAGAGAGAGAGAGAGAGAGAGAGAGAG -5'
         ||||*||||||||||||||||||||||||||||||||||
TTS: 5'- GAGAAAGAGAGAGAGAGAGAGAGAGAGAGAGAGAGAGAG -3'
     3'- CTCTTTCTCTCTCTCTCTCTCTCTCTCTCTCTCTCTCTC -5'

chr1      109656031           109656070
TFO: 3'- GAGAGAGAGAGAGAGAGAGAGAGAGAGAGAGAGAGAGAG -5'
         ||||||*||||||||||||||||||||||||||||||||
TTS: 5'- GAGAGAAAGAGAGAGAGAGAGAGAGAGAGAGAGAGAGAG -3'
     3'- CTCTCTTTCTCTCTCTCTCTCTCTCTCTCTCTCTCTCTC -5'

chr1      109656029           109656068
TFO: 3'- GAGAGAGAGAGAGAGAGAGAGAGAGAGAGAGAGAGAGAG -5'
         ||||||||*||||||||||||||||||||||||||||||
TTS: 5'- GAGAGAGAAAGAGAGAGAGAGAGAGAGAGAGAGAGAGAG -3'
     3'- CTCTCTCTTTCTCTCTCTCTCTCTCTCTCTCTCTCTCTC -5'

chr1      109656027           109656066
TFO: 3'- GAGAGAGAGAGAGAGAGAGAGAGAGAGAGAGAGAGAGAG -5'
         ||||||||||*||||||||||||||||||||||||||||
TTS: 5'- GAGAGAGAGAAAGAGAGAGAGAGAGAGAGAGAGAGAGAG -3'
     3'- CTCTCTCTCTTTCTCTCTCTCTCTCTCTCTCTCTCTCTC -5'

chr1      109656025           109656064
TFO: 3'- GAGAGAGAGAGAGAGAGAGAGAGAGAGAGAGAGAGAGAG -5'
         ||||||||||||*||||||||||||||||||||||||||
TTS: 5'- GAGAGAGAGAGAAAGAGAGAGAGAGAGAGAGAGAGAGAG -3'
     3'- CTCTCTCTCTCTTTCTCTCTCTCTCTCTCTCTCTCTCTC -5'

chr1      109656023           109656062
TFO: 3'- GAGAGAGAGAGAGAGAGAGAGAGAGAGAGAGAGAGAGAG -5'
         ||||||||||||||*||||||||||||||||||||||||
TTS: 5'- GAGAGAGAGAGAGAAAGAGAGAGAGAGAGAGAGAGAGAG -3'
     3'- CTCTCTCTCTCTCTTTCTCTCTCTCTCTCTCTCTCTCTC -5'

chr1      109656021           109656060
TFO: 3'- GAGAGAGAGAGAGAGAGAGAGAGAGAGAGAGAGAGAGAG -5'
         ||||||||||||||||*||||||||||||||||||||||
TTS: 5'- GAGAGAGAGAGAGAGAAAGAGAGAGAGAGAGAGAGAGAG -3'
     3'- CTCTCTCTCTCTCTCTTTCTCTCTCTCTCTCTCTCTCTC -5'

chr1      109656019           109656058
TFO: 3'- GAGAGAGAGAGAGAGAGAGAGAGAGAGAGAGAGAGAGAG -5'
         ||||||||||||||||||*||||||||||||||||||||
TTS: 5'- GAGAGAGAGAGAGAGAGAAAGAGAGAGAGAGAGAGAGAG -3'
     3'- CTCTCTCTCTCTCTCTCTTTCTCTCTCTCTCTCTCTCTC -5'

chr1      109656017           109656056
TFO: 3'- GAGAGAGAGAGAGAGAGAGAGAGAGAGAGAGAGAGAGAG -5'
         ||||||||||||||||||||*||||||||||||||||||
TTS: 5'- GAGAGAGAGAGAGAGAGAGAGAAAGAGAGAGAGAGAGAG -3'
     3'- CTCTCTCTCTCTCTCTCTCTTTCTCTCTCTCTCTCTCTC -5'
```

```
chr1      109656015          109656054
TFO: 3'- GAGAGAGAGAGAGAGAGAGAGAGAGAGAGAGAGAGAGAG -5'
         ||||||||||||||||||||||*||||||||||||||||
TTS: 5'- GAGAGAGAGAGAGAGAGAGAGAAAGAGAGAGAGAGAGAG -3'
     3'- CTCTCTCTCTCTCTCTCTCTCTTTCTCTCTCTCTCTCTC -5
chr1      109656014          109656052
TFO: 3'- AGAGAGAGAGAGAGAGAGAGAGAGAGAGAGAGAGAGAG -5'
         |||||||||||||||||||||||*||||||||||||||
TTS: 5'- AGAGAGAGAGAGAGAGAGAGAGAAAGAGAGAGAGAGAG -3'
     3'- TCTCTCTCTCTCTCTCTCTCTCTTTCTCTCTCTCTCTC -5'

chr1      109656014          109656050
TFO: 3'- AGAGAGAGAGAGAGAGAGAGAGAGAGAGAGAGAGAG -5'
         |||||||||||||||||||||||*||||||||||||
TTS: 5'- AGAGAGAGAGAGAGAGAGAGAGAAAGAGAGAGAGAG -3'
     3'- TCTCTCTCTCTCTCTCTCTCTCTTTCTCTCTCTCTC -5'
```

2. **Gene Symbol: PROX1**

Promoter region (1kb upstream): chr1:214160843-214161843

Potential Triplex Site (PTS):

```
chr1      214161764          214161799
TFO: 3'- GAGAGAGAGAGAGAGAGAGAGAGAGAGAGAGAGAG -5'
         ||||*||||||||||||||||||||||||||||||
TTS: 5'- GAGAtAGAGAGAGAGAGAGAGAGAGAGAGAGAGAG -3'
     3'- CTCTATCTCTCTCTCTCTCTCTCTCTCTCTCTCTC -5'

chr1      214161762          214161799
TFO: 3'- GAGAGAGAGAGAGAGAGAGAGAGAGAGAGAGAGAGAG -5'
         ||||||*||||||||||||||||||||||||||||||
TTS: 5'- GAGAGAtAGAGAGAGAGAGAGAGAGAGAGAGAGAGAG -3'
     3'- CTCTCTATCTCTCTCTCTCTCTCTCTCTCTCTCTCTC -5'

chr1      214161760          214161799
TFO: 3'- GAGAGAGAGAGAGAGAGAGAGAGAGAGAGAGAGAGAGAG -5'
         ||||||||*||||||||||||||||||||||||||||||
TTS: 5'- GAGAGAGAtAGAGAGAGAGAGAGAGAGAGAGAGAGAGAG -3'
     3'- CTCTCTCTATCTCTCTCTCTCTCTCTCTCTCTCTCTCTC -5'


chr1      214161758          214161800
TFO: 3'- GAGAGAGAGAGAGAGAGAGAGAGAGAGAGAGAGAGAGAGttG -5'
         ||||||||||*||||||||||||||||||||||||||||**|
TTS: 5'- GAGAGAGAGAtAGAGAGAGAGAGAGAGAGAGAGAGAGAGAGG -3'
     3'- CTCTCTCTCTATCTCTCTCTCTCTCTCTCTCTCTCTCTCTCC -5'

chr1      214161756          214161795
TFO: 3'- GAGAGAGAGAGAGAGAGAGAGAGAGAGAGAGAGAGAGAG -5'
         ||||||||||||*||||||||||||||||||||||||||
TTS: 5'- GAGAGAGAGAGAtAGAGAGAGAGAGAGAGAGAGAGAGAG -3'
     3'- CTCTCTCTCTCTATCTCTCTCTCTCTCTCTCTCTCTCTC -5'

chr1      214161752          214161793
TFO: 3'- AGGAGAGAGAGAGAGAGAGAGAGAGAGAGAGAGAGAGAGAG -5'
         |*||||||||||||||*||||||||||||||||||||||||
TTS: 5'- AAGAGAGAGAGAGAGAtAGAGAGAGAGAGAGAGAGAGAGAG -3'
     3'- TTCTCTCTCTCTCTCTATCTCTCTCTCTCTCTCTCTCTCTC -5'

chr1      214161750          214161791
TFO: 3'- AGGAGAGAGAGAGAGAGAGAGAGAGAGAGAGAGAGAGAGAG -5'
         |**|||||||||||||||*||||||||||||||||||||||
TTS: 5'- AAAAGAGAGAGAGAGAGAtAGAGAGAGAGAGAGAGAGAGAG -3'
     3'- TTTTCTCTCTCTCTCTCTATCTCTCTCTCTCTCTCTCTCTC -5'
```

chr1 214161748 214161789

```
TFO: 3'- AGGAGAGAGAGAGAGAGAGAGAGAGAGAGAGAGAGAGAGAG -5'
         |**|*|||||||||||||||*||||||||||||||||||||
TTS: 5'- AAAAAAGAGAGAGAGAGAGAtAGAGAGAGAGAGAGAGAGAG -3'
     3'- TTTTTTCTCTCTCTCTCTCTATCTCTCTCTCTCTCTCTCTC -5'
```

chr1 214161749 214161787

```
TFO: 3'- AGAGAGAGAGAGAGAGAGAGAGAGAGAGAGAGAGAGAG -5'
         |*|*||||||||||||||*||||||||||||||||||
TTS: 5'- AAAAAGAGAGAGAGAGAGAtAGAGAGAGAGAGAGAGAG -3'
     3'- TTTTTCTCTCTCTCTCTCTATCTCTCTCTCTCTCTCTC -5'
```

chr1 214161749 214161785

```
TFO: 3'- AGAGAGAGAGAGAGAGAGAGAGAGAGAGAGAGAGAG -5'
         |*|*||||||||||||||*||||||||||||||||
TTS: 5'- AAAAAGAGAGAGAGAGAGAtAGAGAGAGAGAGAGAG -3'
     3'- TTTTTCTCTCTCTCTCTCTATCTCTCTCTCTCTCTC -5'
```

3. Gene: BCL9

Promoter region (1kb upstream): chr1:147012270-147013270

Potential Triplex Sites (PTS):

chr1 147012921 147012956

```
TFO: 3'- GAGAGAGAGAGAGAGAGAGAGAGAGAGAGAGAGAG -5'
         |||||||||||||||||||||||||||||*|*|*|
TTS: 5'- GAGAGAGAGAGAGAGAGAGAGAGAGAGAGtGtGtG -3'
     3'- CTCTCTCTCTCTCTCTCTCTCTCTCTCTCACACAC -5'
```

chr1 147012919 147012956

```
TFO: 3'- GAGAGAGAGAGAGAGAGAGAGAGAGAGAGAGAGAGAG -5'
         |||||||||||||||||||||||||||||||*|*|*|
TTS: 5'- GAGAGAGAGAGAGAGAGAGAGAGAGAGAGAGtGtGtG -3'
     3'- CTCTCTCTCTCTCTCTCTCTCTCTCTCTCTCACACAC -5'
```

chr1 147012918 147012956

```
TFO: 3'- AGAGAGAGAGAGAGAGAGAGAGAGAGAGAGAGAGAGAG -5'
         ||||||||||||||||||||||||||||||||*|*|*|
TTS: 5'- AGAGAGAGAGAGAGAGAGAGAGAGAGAGAGAGtGtGtG -3'
     3'- TCTCTCTCTCTCTCTCTCTCTCTCTCTCTCTCACACAC -5'
```

chr1 147012915 147012954

```
TFO: 3'- GAGAGAGAGAGAGAGAGAGAGAGAGAGAGAGAGAGAGAG -5'
         ||*||||||||||||||||||||||||||||||||*|*|
TTS: 5'- GAcAGAGAGAGAGAGAGAGAGAGAGAGAGAGAGAGtGtG -3'
     3'- CTGTCTCTCTCTCTCTCTCTCTCTCTCTCTCTCTCACAC -5'
```

chr1 147012913 147012952

```
TFO: 3'- GAGAGAGAGAGAGAGAGAGAGAGAGAGAGAGAGAGAGAG -5'
         ||||*||||||||||||||||||||||||||||||||*|
TTS: 5'- GAGAcAGAGAGAGAGAGAGAGAGAGAGAGAGAGAGAGtG -3'
     3'- CTCTGTCTCTCTCTCTCTCTCTCTCTCTCTCTCTCTCAC -5'
```

chr1 147012911 147012950

```
TFO: 3'- GAGAGAGAGAGAGAGAGAGAGAGAGAGAGAGAGAGAGAG -5'
         ||||||*||||||||||||||||||||||||||||||||
TTS: 5'- GAGAGAcAGAGAGAGAGAGAGAGAGAGAGAGAGAGAGAG -3'
     3'- CTCTCTGTCTCTCTCTCTCTCTCTCTCTCTCTCTCTCTC -5'
```

chr1 147012909 147012948

```
TFO: 3'- GAGAGAGAGAGAGAGAGAGAGAGAGAGAGAGAGAGAGAG -5'
         ||||||||*||||||||||||||||||||||||||||||
TTS: 5'- GAGAGAGAcAGAGAGAGAGAGAGAGAGAGAGAGAGAGAG -3'
     3'- CTCTCTCTGTCTCTCTCTCTCTCTCTCTCTCTCTCTCTC -5'
```

```
chr1      147012907            147012946
TFO: 3'- GAGAGAGAGAGAGAGAGAGAGAGAGAGAGAGAGAGAGAG -5'
         ||||||||||*||||||||||||||||||||||||||||
TTS: 5'- GAGAGAGAGAcAGAGAGAGAGAGAGAGAGAGAGAGAGAG -3'
     3'- CTCTCTCTCTGTCTCTCTCTCTCTCTCTCTCTCTCTCTC -5'

chr1      147012905            147012944
TFO: 3'- GAGAGAGAGAGAGAGAGAGAGAGAGAGAGAGAGAGAGAG -5'
         ||||||||||||*||||||||||||||||||||||||||
TTS: 5'- GAGAGAGAGAGAcAGAGAGAGAGAGAGAGAGAGAGAGAG -3'
     3'- CTCTCTCTCTCTGTCTCTCTCTCTCTCTCTCTCTCTCTC -5'

chr1      147012903            147012942
TFO: 3'- GAGAGAGAGAGAGAGAGAGAGAGAGAGAGAGAGAGAGAG -5'
         ||||||||||||||*||||||||||||||||||||||||
TTS: 5'- GAGAGAGAGAGAGAcAGAGAGAGAGAGAGAGAGAGAGAG -3'
     3'- CTCTCTCTCTCTCTGTCTCTCTCTCTCTCTCTCTCTCTC -5'

chr1      147012901            147012940
TFO: 3'- GAGAGAGAGAGAGAGAGAGAGAGAGAGAGAGAGAGAGAG -5'
         ||||||||||||||||*||||||||||||||||||||||
TTS: 5'- GAGAGAGAGAGAGAGAcAGAGAGAGAGAGAGAGAGAGAG -3'
     3'- CTCTCTCTCTCTCTCTGTCTCTCTCTCTCTCTCTCTCTC -5'

chr1      147012899            147012938
TFO: 3'- GAGAGAGAGAGAGAGAGAGAGAGAGAGAGAGAGAGAGAG -5'
         ||||||||||||||||||*||||||||||||||||||||
TTS: 5'- GAGAGAGAGAGAGAGAGAcAGAGAGAGAGAGAGAGAGAG -3'
     3'- CTCTCTCTCTCTCTCTCTGTCTCTCTCTCTCTCTCTCTC -5'

chr1      147012897            147012936
TFO: 3'- GAGAGAGAGAGAGAGAGAGAGAGAGAGAGAGAGAGAGAG -5'
         ||||||||||||||||||||*||||||||||||||||||
TTS: 5'- GAGAGAGAGAGAGAGAGAGAcAGAGAGAGAGAGAGAGAG -3'
     3'- CTCTCTCTCTCTCTCTCTCTGTCTCTCTCTCTCTCTCTC -5'

chr1      147012897            147012934
TFO: 3'- GAGAGAGAGAGAGAGAGAGAGAGAGAGAGAGAGAGAG -5'
         ||||||||||||||||||||*||||||||||||||||
TTS: 5'- GAGAGAGAGAGAGAGAGAGAcAGAGAGAGAGAGAGAG -3'
     3'- CTCTCTCTCTCTCTCTCTCTGTCTCTCTCTCTCTCTC -5'

chr1      147012897            147012932
TFO: 3'- GAGAGAGAGAGAGAGAGAGAGAGAGAGAGAGAGAG -5'
         ||||||||||||||||||||*||||||||||||||
TTS: 5'- GAGAGAGAGAGAGAGAGAGAcAGAGAGAGAGAGAG -3'
     3'- CTCTCTCTCTCTCTCTCTCTGTCTCTCTCTCTCTC -5'
```
