# Supplementary figures and images for: Genome-wide computational analysis of potential long noncoding RNA mediated DNA:DNA:RNA triplexes in the human genome
Source: J Transl Med. 2017 Sep 2;15:186. doi: 10.1186/s12967-017-1282-9 (PMC7670996; doi:10.1186/s12967-017-1282-9)

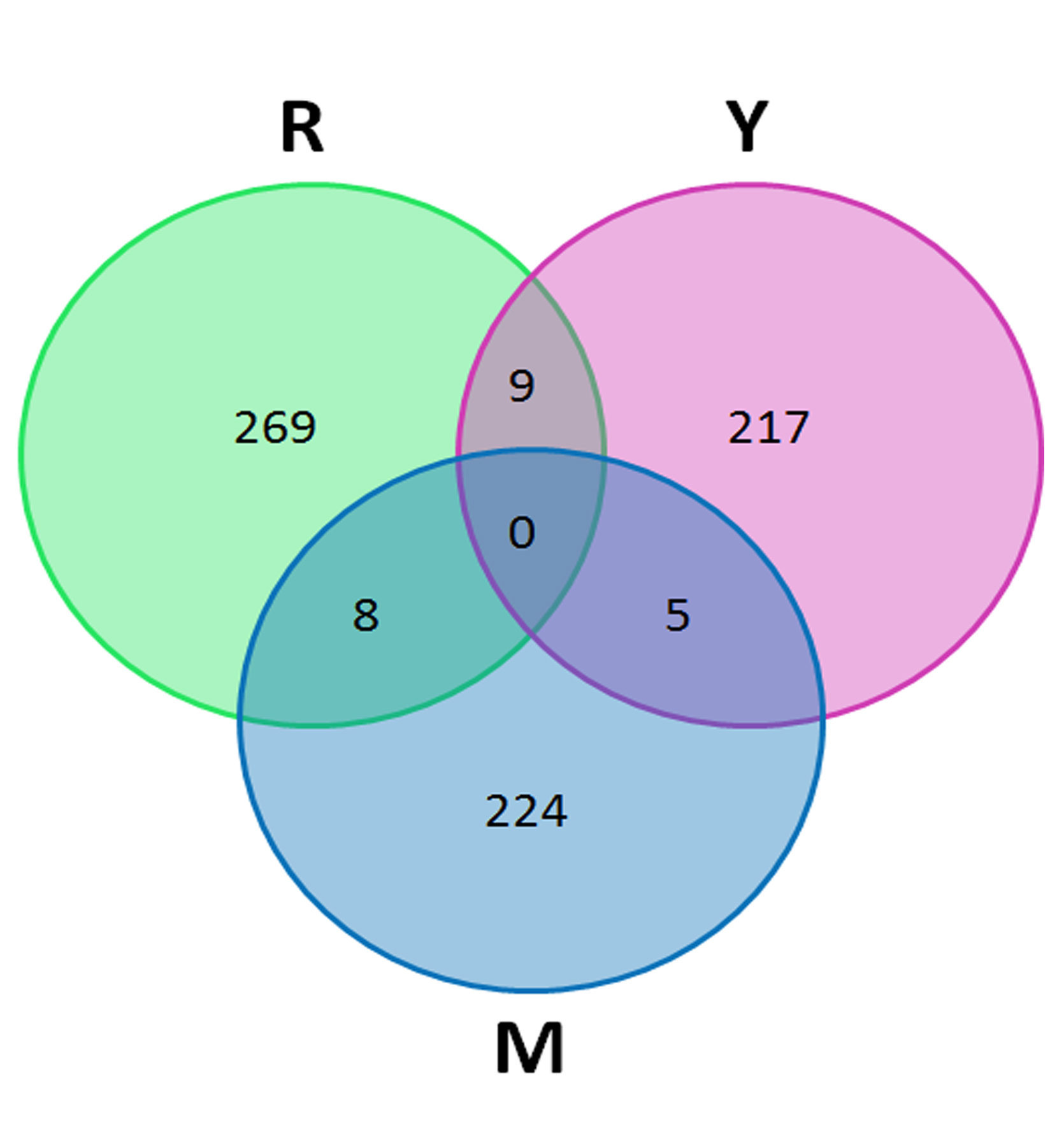

Supplement: Supplementary file 8 — Additional file 8. Venn diagram representing the overlap of lncRNAs forming the three types of motifs (R, Y & M). [file 12967_2017_1282_MOESM8_ESM.jpg]

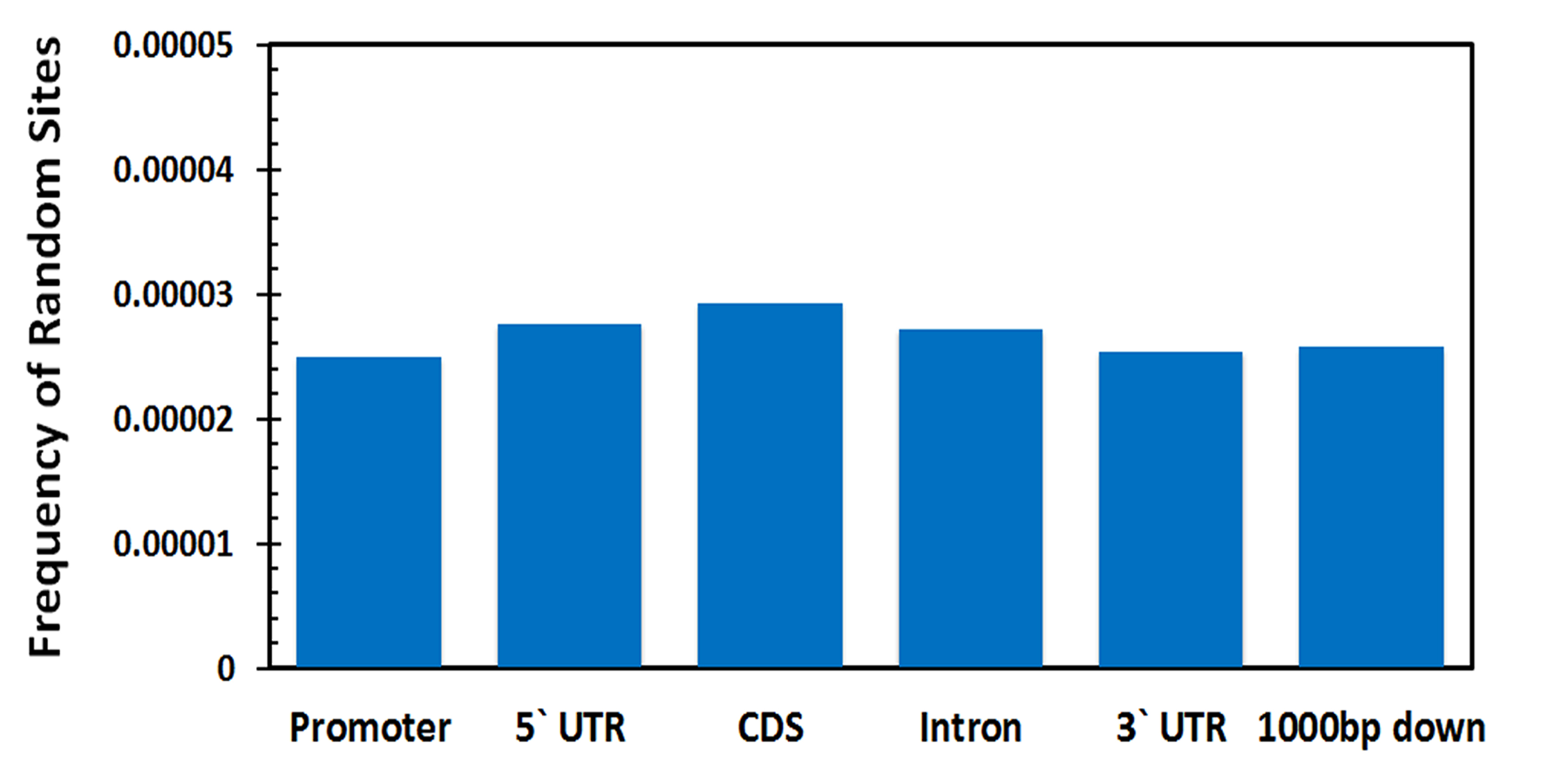

Supplement: Supplementary file 9 — Additional file 9. Distribution of random genomic loci across the Refseq genes and associated genomic features including Promoter, 5′ UTR, CDS, Intron, 3′ UTR and 1000 bases downstream. [file 12967_2017_1282_MOESM9_ESM.jpg]

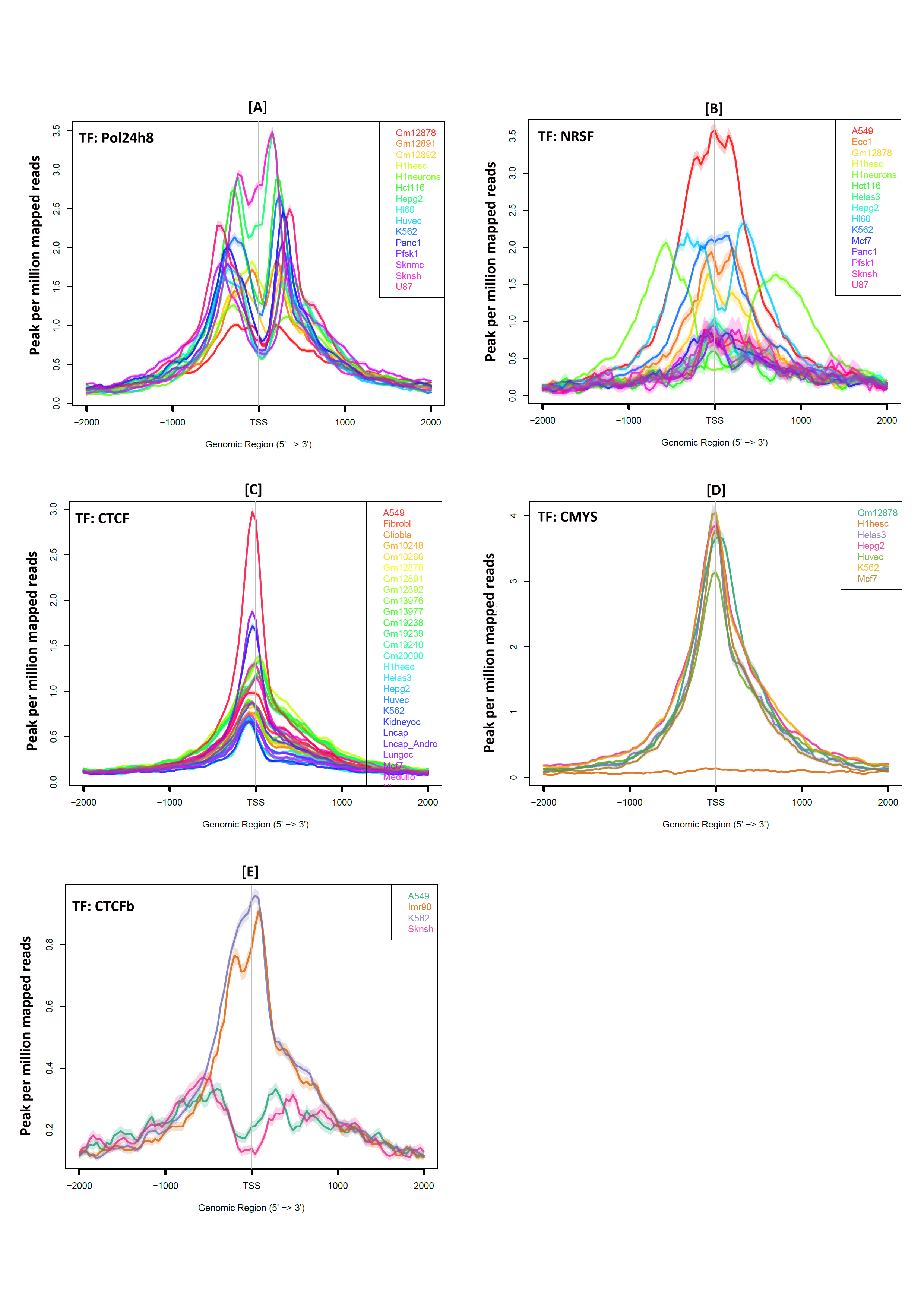

Supplement: Supplementary file 12 — Additional file 12. Distribution of Transcription factors across the Transcription Start Site (TSS) (A) Pol24h8 (B) NRSF (C) CTCF (D) CMYS (E) CTCFB. [file 12967_2017_1282_MOESM12_ESM.jpg]

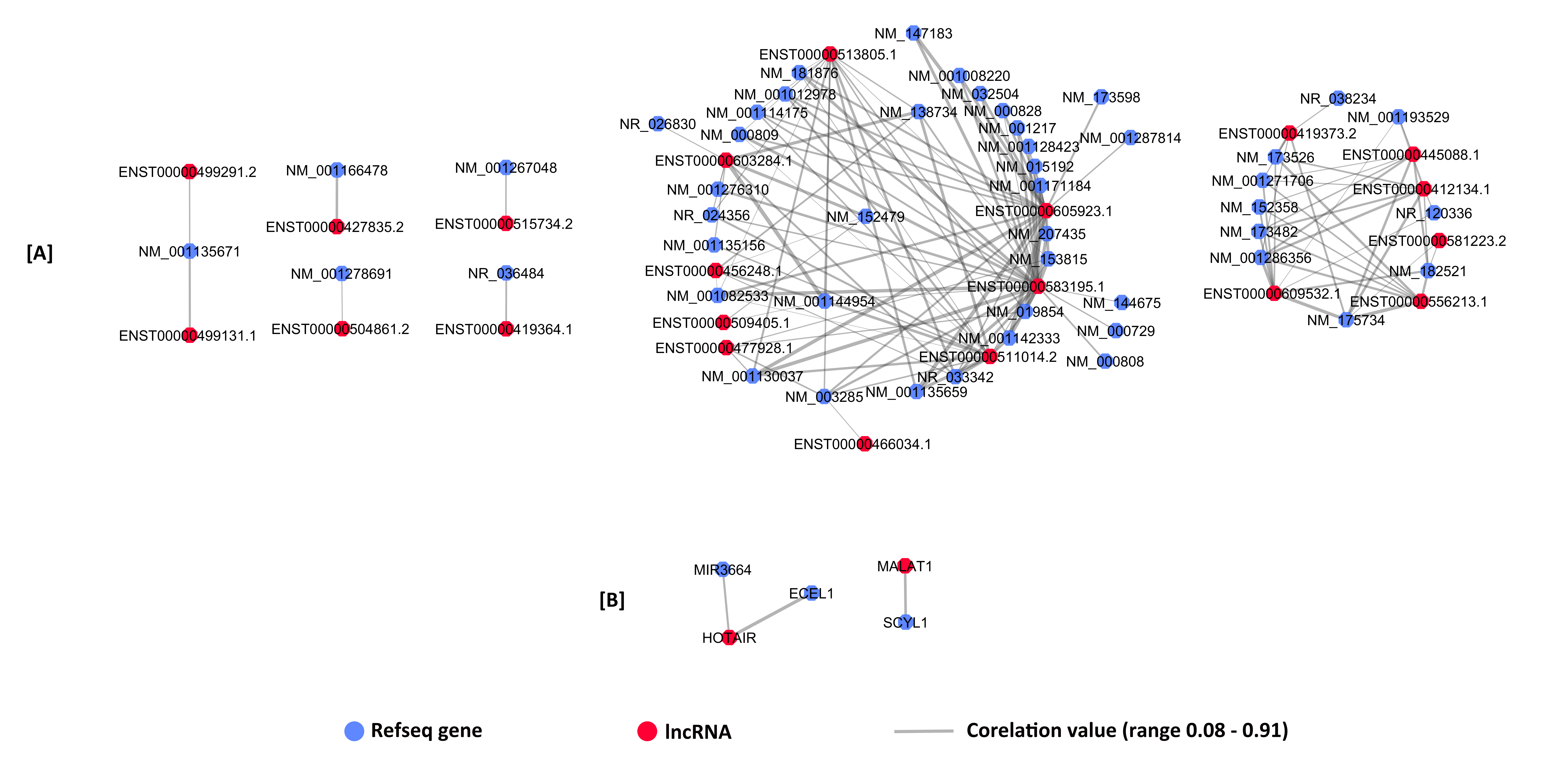

Supplement: Supplementary file 24 — Additional file 24. Correlation network constructed using Cytoscape for 23 lncRNAs and 51 Refseq genes. The Refseq genes are represented by blue node, lncRNAs by red node and the edge (grey) represents the correlation value. The thickness of the edge represents the value ranging from 0.08 to 0.91, thicker the line higher the correlation. [A] The network for lncRNAs forming triplex structure with promoters of Refseq gene predicted with these parameters (-l 35 -e 10 -g 20 -m R/Y/M -fm 0 -of 1 -fr off). [B] Interaction network for HOTAIR and MALAT1 lncRNAs, which are known to form triplex structure from previous literatures [14] and were predicted in our analysis with these parameters (-l 35/40 -e 20/20 -g 20 -m R/Y/M -fm 0 -of 1 -fr off). [file 12967_2017_1282_MOESM24_ESM.jpg]
