# Supplementary material for: Genome-wide computational analysis of potential long noncoding RNA mediated DNA:DNA:RNA triplexes in the human genome
Source: J Transl Med. 2017 Sep 2;15:186. doi: 10.1186/s12967-017-1282-9 (PMC7670996; doi:10.1186/s12967-017-1282-9)
Supplement: Supplementary file 10 — Additional file 10. Conservation of Housekeeping and Tissue specific genes across the PTS, tested by Chi-square test. [file 12967_2017_1282_MOESM10_ESM.pdf]

<table>
<tr><th></th><th>Housekeeping genes+</th><th>Tissue specific genes</th><td rowspan="3">Chi-square 932054.574
and P-value 0</td></tr>
<tr><td>PTS+</td><td>577672</td><td>0</td></tr>
<tr><td>PTS-</td><td>345666</td><td>1054696</td></tr>
</table>
