# Supplementary material for: Genome-wide computational analysis of potential long noncoding RNA mediated DNA:DNA:RNA triplexes in the human genome
Source: J Transl Med. 2017 Sep 2;15:186. doi: 10.1186/s12967-017-1282-9 (PMC7670996; doi:10.1186/s12967-017-1282-9)
Supplement: Supplementary file 11 — Additional file 11. The results of Chi-square test for testing mapping significance of the three transcription factors mapping to PTS. [A] Chi-square test to check the mapping significance of UTA Transcription Factor Binding sites (UTA TFBS) with Potential Triplex Sites. [B] Chi-square test to check the mapping significance of HAIB Transcription Factor Binding sites (HAIB TFBS) with Potential Triplex Sites. [C] Chi-square test to check the mapping significance of SYDH Transcription Factor Binding sites (SYDH TFBS) with Potential Triplex Sites. [file 12967_2017_1282_MOESM11_ESM.pdf]

[A] Chi-square test to check the mapping significance of UTA Transcription Factor Binding sites (UTA TFBS) with Potential Triplex Sites.

Cell Line and TF	A	B	C	D	Chi square	p value
Fibrobl Ctcf Rep1	828303	2985288	9875771	3123471902	51320569.21	0
Gm10248 Ctcf Rep1	848380	2965211	9317814	3124029859	56809037.75	0
Gm10266 Ctcf Rep1	959394	2854197	9835167	3123512506	68557480.28	0
Gm13976 Ctcf Rep1	539629	3273962	7147186	3126200487	30204266.56	0
Gm20000 Ctcf Rep1	647438	3166153	7339443	3126008230	42046822.83	0
Kidneyoc Ctcf Rep1	1810641	2002950	7933129	3125414544	274358812.5	0
Lncap Ctcf Andro Rep1	836269	2977322	9427522	3123920151	54636350.8	0
Lncap Ctcf Rep1	808834	3004757	10709302	3122638371	45341647.79	0
LungOC Ctcf Rep1	1780946	2032645	7875427	3125472246	267801719.7	0
Mcf7 Cmyc Estro Rep1	232706	3580885	2978782	3130368891	13439692.78	0
Pancreas OC Ctcf Rep1	840534	2973057	9610934	3123736739	54185643.79	0
Progfib Ctcf Rep1	1181731	2631860	8992654	3124355019	111053480.8	0
Spleen OC Ctcf Rep1	926643	2886948	11549379	3121798294	55065143.27	0

A: Unique number of bases of mapping between PTS and UTA TFBS; B: Unique number of bases of PTS not mapping to UTA TFBS;

C: Unique number of bases of UTA TFBS not mapping to PTS domains and D: Total unique bases of (A+B+C) minus from unique bases of human genome

[B] Chi-square test to check the mapping significance of HAIB Transcription Factor Binding sites (HAIB TFBS) with Potential Triplex Sites.

Cell Line and TF	A	B	C	D	Chi square	p value
A549 Gr	9928	3803663	274903	3133072770	265505.313	0
A549 Sin3ak20	507496	3306095	5833870	3127513803	32508534.5	0
Ecc1 Nrsf	501691	3311900	4315837	3129031836	42096699.3	0
Ecc1 Nrsf	493407	3320184	4568413	3128779260	38693319.3	0
Gm12878 Egr1	84624	3728967	873669	3132474004	5988424.16	0
Gm12878 Ets1	90869	3722722	455989	3132891684	12257049.2	0
Gm12878 tIrf4sc6059	1310025	2503566	1.4E+07	3119179210	89154580.6	0
Gm12878 Nrsf	305462	3508129	3173665	3130174008	21505367.2	0
Gm12878 Nrsf	103553	3710038	1375453	3131972220	5768702.72	0
Gm12878 Nrsf	48350	3765241	967838	3132379835	1799743.21	0
Gm12878 P300	718564	3095027	8052867	3125294806	47186951.2	0
Gm12878 P300	429062	3384529	2488236	3130859437	51166460.2	0
Gm12878 Pbx3	444551	3369040	4080486	3129267187	35136965.4	0
Gm12878 Pou2f2	486783	3326808	6630436	3126717237	26515583	0
Gm12878 Rxra	284871	3528720	2707915	3130639758	21787235.3	0
Gm12878 Zbtb33	554634	3258957	5478853	3127868820	40968389.5	0
Gm12878 Zbtb33	620675	3192916	5420287	3127927386	51386910.4	0

H1hesc Bcl11a	56026	3757565	624368	3132723305	3689143.66	0
H1hesc Bcl11a	37802	3775789	347609	3133000064	2978909.83	0
H1hesc Egr1	147738	3665853	1148619	3132199054	13578617.8	0
H1hesc Fosl1sc183	38203	3775388	163184	3133184489	5893027.01	0
H1hesc Nrsf	383229	3430362	4207249	3129140424	25626251.6	0
H1hesc Nrsf	430420	3383171	4853472	3128494201	28069476.6	0
H1hesc Pou5f1sc9081	46957	3766634	554031	3132793642	2929065.37	0
H1hesc Rxra	46450	3767141	299677	3133047996	5042095.51	0
H1hesc Six5	83950	3729641	815224	3132532449	6290274.56	0
H1hesc Sp2	66810	3746781	590999	3132756674	5456904.96	0
Hct116 Nrsf	221560	3592031	1236982	3132110691	27290902	0
Hct116 Nrsf	299348	3514243	1613101	3131734572	38017773.4	0
Hct116 Zbtb33	72494	3741097	461901	3132885772	7956612.08	0
Helas3 Gabp	300573	3513018	3216640	3130131033	20581414.1	0
Hepg2 Gabp	218312	3595279	2645711	3130701962	13284425.9	0
Hepg2 Nrsf	81115	3732476	837933	3132509740	5736883.11	0
Hepg2 Nrsf	85007	3728584	1275298	3132072375	4208521.64	0
Hepg2 Nrsf	261357	3552234	2028842	3131318831	24062527.8	0
Hepg2 Sp1	124113	3689478	1348439	3131999234	8372980.14	0

Hepg2 Sp2	164910	3648681	1360022	3131987651	14367018.2	0
Hepg2 Zbtb33	281046	3532545	2838748	3130508925	20313806.6	0
Hepg2 Zbtb33	73211	3740380	440859	3132906814	8442803.26	0
Hepg2 Zbtb33	106796	3706795	598220	3132749453	13114179.2	0
Hl60 Nrsf	327816	3485775	2301165	3131046508	33041413.5	0
K562 Six5	503306	3310285	4732954	3128614719	38908493.9	0
K562 Sp2sc643	146185	3667406	1416679	3131930994	10976642.1	0
K562 Srf	51046	3762545	475189	3132872484	3977349.33	0
K562 Zbtb33	532285	3281306	5153259	3128194414	40057437.5	0
K562 Zbtb33	840969	2972622	6074295	3127273378	82739801.4	0
Mcf7 Nrsf	353944	3459647	3613150	3129734523	25337355.7	0
Mcf7 Nrsf	430842	3382749	3430855	3129916818	38780009.7	0
Panc1 Nrsf	213631	3599960	2476653	3130871020	13559211.1	0
Panc1 Nrsf	75026	3738565	1026257	3132321416	4062275.31	0
Panc1 Nrsf	331866	3481725	1733311	3131614362	43290203	0
Panc1 Nrsf	308857	3504734	2351253	3130996420	28944948.7	0
Pfsk1 Nrsf	83397	3730194	1171837	3132175836	4399886.78	0
Pfsk1 Nrsf	171907	3641684	2421034	3130926639	9053377.23	0
Sknmc Foxp2	116798	3696793	832927	3132514746	11601310.3	0

Sknmc Foxp2	1353814	2459777	8092817	3125254856	157573316	0
Sknsh Nrsf	16333	3797258	339278	3133008395	585650.091	0
U87 Nrsf	88741	3724850	715135	3132632538	7893761.45	0
U87 Nrsf	107581	3706010	797142	3132550531	10324915.6	0
U87 Nrsf	264232	3549359	2017580	3131330093	24692872.7	0
U87 Nrsf	216176	3597415	1927131	3131420542	17539862.1	0

A: Unique number of bases of mapping between PTS and HAIB TFBS; B: Unique number of bases of PTS not mapping to HAIB TFBS;

C: Unique number of bases of HAIB TFBS not mapping to PTS domains and D: Total unique bases of (A+B+C) minus from unique bases of human genome

[C} Chi-square test to check the mapping significance of SYDH Transcription Factor Binding sites (SYDH TFBS) with Potential Triplex Sites.

Cell Line and TF	A	B	C	D	Chi square	p value
Gm08714 Znf274	94521	3719070	927148	3132420525	7016658.963	0
Gm12878 Brca1a300	351576	3462015	3481582	3129866091	25891353.5	0
H1hesc Znf274m01	161118	3652473	1132269	3132215404	16216307.89	0
Hek293t Znf263	3342560	471031	29277562	3104070111	278341112.3	0
Helas3 Bdp1	54832	3758759	242488	3133105185	8220001.598	0
Helas3 Brf1	47935	3765656	142378	3133205295	9848999.957	0
Helas3 Brf2	63498	3750093	47351	3133300322	29832485.76	0
Helas3 Brg1	12658	3800933	72271	3133275402	1528633.868	0
Helas3 Gcn5	183648	3629943	688845	3132658828	31479756	0
Helas3 Prdm19115	311597	3501994	3882310	3129465363	18473566.55	0
Helas3 Rpc155	240241	3573350	1547879	3131799794	26120502.84	0
Helas3 Spt20	25175	3788416	178949	3133168724	2507266.128	0
Helas3 Tcf7l2	252045	3561546	1525711	3131821962	28945539.84	0
Helas3 Tf3c110	335063	3478528	2301191	3131046482	34436124.06	0
Helas3 Znf274	52039	3761552	157141	3133190532	10559491.41	0
Hepg2 Erra	11289	3802302	186651	3133161022	507951.292	0
Hepg2 Jund	3421388	392203	22691400	3110656273	365440095.6	0

Imr90 Ctcfb	1561651	2251940	17780727	3115566946	101367261.2	0
K562 Atf3	39999	3773592	287407	3133060266	3945499.288	0
K562 Gata1	106163	3707428	677893	3132669780	11630692.36	0
K562 Pol2s2	3204279	609312	28017762	3105329911	267131156.4	0
K562 Pol3	5621	3807970	44867	3133302806	504244.235	0
K562 Rpc155	63488	3750103	426146	3132921527	6654707.268	0
K562 Sirt6	85663	3727928	228440	3133119233	19072547.03	0
K562 Stat1	62518	3751073	445357	3132902316	6214904.117	0
K562 Tr4	29745	3783846	135548	3133212125	4349506.27	0
K562Xrcc4 Std	19686	3793905	1618	3133346055	14943225.05	0
K562 Znf263	166581	3647010	1616871	3131730802	12490759.56	0
K562 Znf274	25988	3787603	257584	3133090089	1910091.65	0
Nt2d1 Suz12	304277	3509314	3045736	3130301937	22181114.81	0

A: Unique number of bases of mapping between PTS and SYDH TFBS; B: Unique number of bases of PTS not mapping to HAIB TFBS; C: Unique number of bases of HAIB TFBS not mapping to PTS domains and D: Total unique bases of (A+B+C) minus from unique bases of human genome
