# Supplementary material for: Genome-wide computational analysis of potential long noncoding RNA mediated DNA:DNA:RNA triplexes in the human genome
Source: J Transl Med. 2017 Sep 2;15:186. doi: 10.1186/s12967-017-1282-9 (PMC7670996; doi:10.1186/s12967-017-1282-9)
Supplement: Supplementary file 22 — Additional file 22. The results of Chi-square test for testing mapping significance of the Hi-C mapping to PTS. [A] Chi-square test to check the mapping significance of Hi-C interaction domain with Potential Triplex Sites forming purine motif (R). [B] Chi-square test to check the mapping significance of Hi-C interaction domain with Potential Triplex Sites forming pyrimidine motif (Y). [C] Chi-square test to check the mapping significance of Hi-C interaction domain with Potential Triplex Sites forming purine-pyrimidine motif (M). [file 12967_2017_1282_MOESM22_ESM.pdf]

[A] Chi-square test to check the mapping significance of Hi-C interaction domain with Potential Triplex Sites forming purine motif (R).

Hi-C data	A	B	C	D	Chi-square	P value
CCHiC-HeLaS3CCL2p2-G1-0.25FA	80566	68272040	3733025	3065075633	78.516	0
CCHiC-HeLaS3CCL2p2-G1-1FA	90542	76471043	3723049	3056876630	70.452	0
CCHiC-HeLaS3CCL2p2-M-0.25FA	19332	16401766	3794259	3116945907	19.999	0.00000775
CCHiC-HeLaS3CCL2p2-M-1FA	30757	14170082	3782834	3119177591	10609.17	0
CCHiC-HeLaS3CCL2p2-M-98percent	130863	26151746	3682728	3107195927	309190.6	0
CCHiC-HeLaS3-G1mid-R1	29352	25089599	3784239	3108258074	46.263	0
CCHiC-HeLaS3-G1mid-R2	16715	14184124	3796876	3119163549	17.484	0.00002897
CCHiC-HeLaS3-M-R1	40210	34198481	3773381	3099149192	48.434	0
CCHiC-HeLaS3-M-R2	31090	26372254	3782501	3106975419	31.862	2.00E-08
CCHiC-HFF1-M-R1	126330	105952476	3687261	3027395197	55.219	0
CCHiC-HFF1-NS-R1	151462	126894575	3662129	3006453098	59.9	0
CCHiC-K562-M-R1	21482	18279234	3792109	3115068439	26.471	2.70E-07
CCHiC-K562-M-R2	43573	36867079	3770018	3096480594	37.941	0

A: Unique number of bases of mapping between PTS and Hi-C domains; B: Unique number of bases of Hi-C domains not mapping to PTS;

C:Unique number of bases of PTS not mapping to Hi-C domains and D: Total unique bases of A+B+C minus from unique bases of human genome

[B] Chi-square test to check the mapping significance of Hi-C interaction domain with Potential Triplex Sites forming pyrimidine motif (Y).

Hi-C data	A	B	C	D	Chi-square	P value
CCHiC-HeLaS3CCL2p2-G1-0.25FA	82325	68270281	3788326	3065020332	48.98	0
CCHiC-HeLaS3CCL2p2-G1-1FA	55794	76505791	3814857	3056784822	16244.878	0
CCHiC-HeLaS3CCL2p2-M-0.25FA	19776	16401322	3850875	3116889291	11.66	0.00063858
CCHiC-HeLaS3CCL2p2-M-1FA	31501	14169338	3839150	3119121275	11218.995	0
CCHiC-HeLaS3CCL2p2-M-98percent	133789	26148820	3736862	3107141793	319903.48	0
CCHiC-HeLaS3-G1mid-R1	30154	25088797	3840497	3108201816	22.866	0.00000174
CCHiC-HeLaS3-G1mid-R2	17300	14183539	3853351	3119107074	2.806	0.09391227
CCHiC-HeLaS3-M-R1	41223	34197468	3829428	3099093145	24.976	5.80E-07
CCHiC-HeLaS3-M-R2	31762	26371582	3838889	3106919031	20.569	0.00000575
CCHiC-HFF1-M-R1	129239	105949567	3741412	3027341046	21.341	0.00000384
CCHiC-HFF1-NS-R1	154922	126891115	3715729	3006399498	22.252	0.00000239
CCHiC-K562-M-R1	22050	18278666	3848601	3115011947	12.508	0.00040521
CCHiC-K562-M-R2	44596	36866056	3826055	3096424557	19.81	0.00000837

A: Unique number of bases of mapping between PTS and Hi-C domains; B: Unique number of bases of Hi-C domains not mapping to PTS; C:Unique number of bases of PTS not mapping to Hi-C domains and D: Total unique bases of A+B+C minus from unique bases of human genome

[C] Chi-square test to check the mapping significance of Hi-C interaction domain with Potential Triplex Sites forming purine-pyrimidine motif (M).

Hi-C data	A	B	C	D	Chi-square	P value
CCHiC-HeLaS3CCL2p2-G1-0.25FA	100230	68252376	4612406	3064196252	59.808	0
CCHiC-HeLaS3CCL2p2-G1-1FA	112850	76448735	4599786	3055999893	41.668	0
CCHiC-HeLaS3CCL2p2-M-0.25FA	24125	16396973	4688511	3116051655	12.022	0.00052576
CCHiC-HeLaS3CCL2p2-M-1FA	38445	14162394	4674191	3118286234	13810.546	0
CCHiC-HeLaS3CCL2p2-M-98percent	162496	26120113	4550140	3106328515	387099.43	0
CCHiC-HeLaS3-G1mid-R1	36568	25082383	4676068	3107366245	36.353	0
CCHiC-HeLaS3-G1mid-R2	20871	14179968	4691765	3118268660	10.043	0.00152928
CCHiC-HeLaS3-M-R1	50065	34188626	4662571	3098260002	36.857	0
CCHiC-HeLaS3-M-R2	38560	26364784	4674076	3106083844	30.983	3.00E-08
CCHiC-HFF1-M-R1	156948	105921858	4555688	3026526770	37.572	0
CCHiC-HFF1-NS-R1	188468	126857569	4524168	3005591059	30.986	3e-8
CCHiC-K562-M-R1	26735	18273981	4685901	3114174647	20.959	0.00000469
CCHiC-K562-M-R2	54165	36856487	4658471	3095592141	30.044	4.00E-08

A: Unique number of bases of mapping between PTS and Hi-C domains; B: Unique number of bases of Hi-C domains not mapping to PTS; C:Unique number of bases of PTS not mapping to Hi-C domains and D: Total unique bases of A+B+C minus from unique bases of human genome
